# Capture severity, infectious disease processes and sex influence post-release mortality of sockeye salmon bycatch

**DOI:** 10.1093/conphys/cox017

**Published:** 2017-03-28

**Authors:** Amy K. Teffer, Scott G. Hinch, Kristi M. Miller, David A. Patterson, Anthony P. Farrell, Steven J. Cooke, Arthur L. Bass, Petra Szekeres, Francis Juanes

**Affiliations:** 1 Department of Biology, University of Victoria, Victoria, BC V8P 5C2, Canada; 2 Salmon Ecology and Conservation Laboratory, Department of Forest and Conservation Sciences, University of British Columbia, Vancouver, BC V6T 1Z4, Canada; 3 Fisheries and Oceans Canada, Molecular Genetics Section, Pacific Biological Station, Nanaimo, BC V9T 6N7, Canada; 4 Fisheries and Oceans Canada, Cooperative Resource Management Institute, School of Resource and Environmental Management, Simon Fraser University, Burnaby, BC V5A 1S6, Canada; 5 Department of Zoology, Department of Land and Food Systems, University of British Columbia, Vancouver, BC V6T 1Z4, Canada; 6 Fish Ecology and Conservation Physiology Laboratory, Department of Biology and Institute of Environmental Science, Carleton University, Ottawa, ON K1S 5B6, Canada

**Keywords:** Bycatch, fisheries, gene expression, infectious disease, Pacific salmon, temperature

## Abstract

Bycatch is a common occurrence in heavily fished areas such as the Fraser River, British Columbia, where fisheries target returning adult Pacific salmon (*Oncorhynchus* spp.) *en route* to spawning grounds. The extent to which these encounters reduce fish survival through injury and physiological impairment depends on multiple factors including capture severity, river temperature and infectious agents. In an effort to characterize the mechanisms of post-release mortality and address fishery and managerial concerns regarding specific regulations, wild-caught Early Stuart sockeye salmon (*Oncorhynchus nerka*) were exposed to either mild (20 s) or severe (20 min) gillnet entanglement and then held at ecologically relevant temperatures throughout their period of river migration (mid–late July) and spawning (early August). Individuals were biopsy sampled immediately after entanglement and at death to measure indicators of stress and immunity, and the infection intensity of 44 potential pathogens. Biopsy alone increased mortality (males: 33%, females: 60%) when compared with non-biopsied controls (males: 7%, females: 15%), indicating high sensitivity to any handling during river migration, especially among females. Mortality did not occur until 5–10 days after entanglement, with severe entanglement resulting in the greatest mortality (males: 62%, females: 90%), followed by mild entanglement (males: 44%, females: 70%). Infection intensities of *Flavobacterium psychrophilum* and *Ceratonova shasta* measured at death were greater in fish that died sooner. Physiological indicators of host stress and immunity also differed depending on longevity, and indicated anaerobic metabolism, osmoregulatory failure and altered immune gene regulation in premature mortalities. Together, these results implicate latent effects of entanglement, especially among females, resulting in mortality days or weeks after release. Although any entanglement is potentially detrimental, reducing entanglement durations can improve post-release survival.

## Teaser

Among Pacific salmon released from gillnets on their way to spawning grounds, those entangled for the longest amount of time, especially females, were very likely to die before spawning. Fish that died earliest were in poorer health than survivors and showed greater activity of several infectious disease causing microbes.

## Introduction

For wild semelparous Pacific salmon (*Oncorhynchus* spp.), lifetime fitness hinges on the survival and successful migration of adults to spawning grounds where they will deposit gametes prior to natural death (Groot and Margolis, 1991). Pacific salmon productivity is in a state of decline in many natal watersheds, especially at southern range extremes (Hinch *et al*., 2012; Peterman and Dorner, 2012). Pre-spawning and *en route* mortality of adult Pacific salmon have likely contributed to these declines and have been attributed to several factors, including thermal and fisheries stressors encountered during freshwater migration (Gilhousen, 1990; Donaldson *et al*., 2011; Martins *et al*., 2012b; Gale *et al*., 2013; Raby *et al*., 2015). Disease processes are also known to influence the survival of wild salmon but have been notoriously difficult to study due to the logistical constraints inherent in monitoring wild animal populations under natural conditions, especially for highly migratory species (Altizer *et al*., 2011; Miller *et al*., 2014). With regard to adult Pacific salmon, the manner by which fisheries practices, temperature and disease processes interact to influence the mechanisms of premature mortality remains poorly understood (Miller *et al*., 2014).

The intense salmon fisheries of the West Coast of North America yield a strong likelihood of gear encounter by migrating adult salmon *en route* to natal streams, rivers and lakes. Although much of this catch is retained, non-target species are often captured and viable bycatch released back to the water, depending on regulations specific to each fishery. In addition to those released, many fish get trapped in gear but escape during the fishing and landing process, displaying physical signs of entanglement at locations further upriver (Baker and Schindler, 2009; Casselman *et al*., 2016). Depending on the fishery, a proportion of captured and released individuals are assumed to arrive at spawning grounds and this subtotal can be counted towards spawner escapement goals set by management. There are physiological consequences of capture and release or escape from fisheries gear that contribute to post-release impairment and mortality (reviewed in Davis, 2002). Variability in these physiological responses is common within and among species and stocks (Cooke and Suski, 2005), and is associated with the severity of the capture event (Gale *et al*., 2011), the condition of the individual at capture (Davis, 2002; Donaldson *et al*., 2012) and the animal's ability to recover (Farrell *et al*., 2001; Robinson *et al*., 2013). Condition at capture and subsequent recovery are also suspected to be associated with infectious disease processes (Gilhousen, 1990; Raby *et al*., 2015). Stress and injury caused by a gear encounter can provide opportunities for infection (Chopin and Arimoto, 1995; Baker and Schindler, 2009; Baker *et al*., 2013), elicit enhanced immune surveillance and responses by the host (Dhabhar, 2002; Neeman *et al*., 2012) and promote physiological disturbances such as osmoregulatory imbalance that can impair overall host health and resilience (Gale *et al*., 2011; Donaldson *et al*., 2012; Cooke *et al*., 2013). Establishing linkages between physiological and infection-associated variables would aid in developing a clearer understanding of how host–parasite relationships impact the survival of released salmon bycatch and improve mortality estimates.

**Figure 1:**
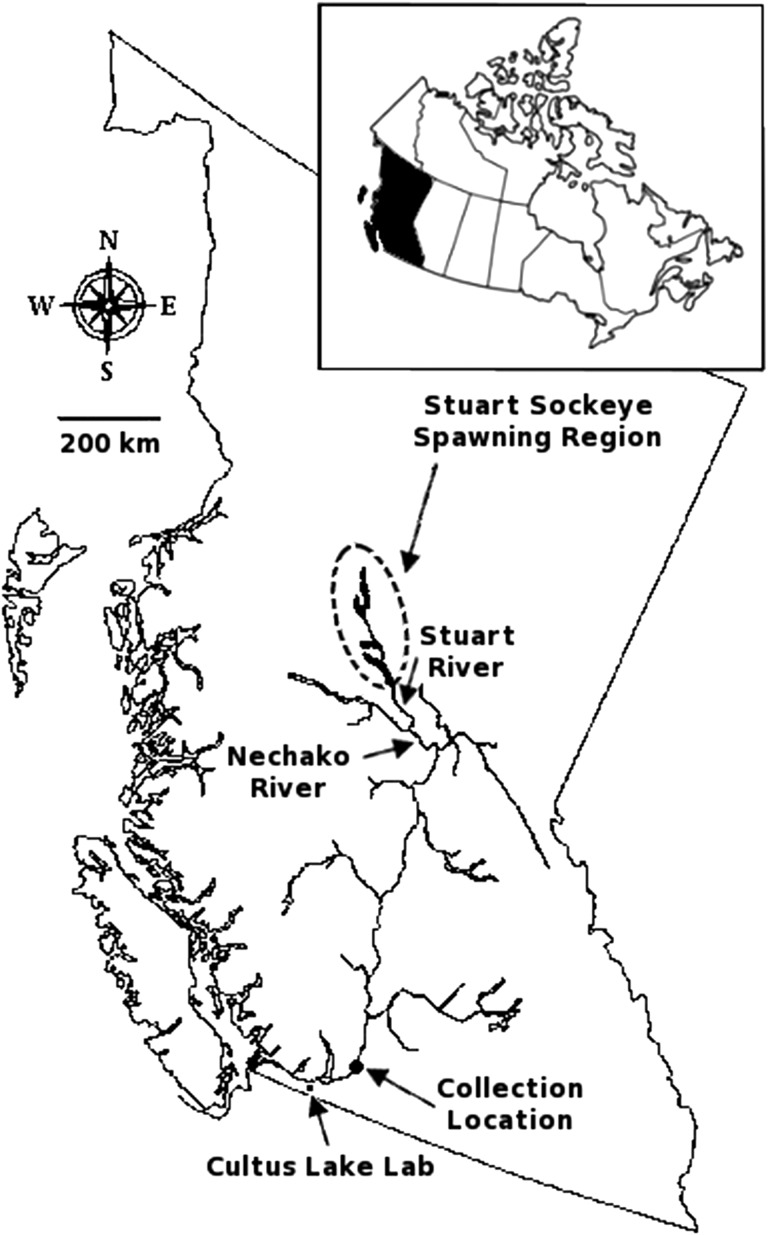
British Columbia, Canada and the Fraser River watershed. Early Stuart sockeye enter the Fraser River in early to mid-July, migrating ~1200 km to spawning grounds (dashed circle) in the interior of the province. Fish pass through the Nechako and Stuart rivers before reaching corridor lakes (shown in black, from north to south: Takla, Trembleur and Stuart). Locations of collection (Yale, BC) and experimental holding (DFO Cultus Lake Salmon Research Lab) are shown.

Environmental factors such as high water temperatures compound the effects of fisheries capture (Gale *et al*., 2013) and have disease-associated consequences, potentially diminishing host (salmon) resilience (Jeffries *et al*., 2012b; Dittmar *et al*., 2014) and altering the productivity of infectious agents prior to (Chiaramonte, 2013; Paull and Johnson, 2014) or following (Thomas and Blanford, 2003; Bettge *et al*., 2009; Kocan *et al*., 2009) infection. One suspected mitigation measure used by Pacific salmon faced with high river temperatures is behavioural thermoregulation, particularly in the lentic components of the migration route (Donaldson *et al*., 2009). By residing in the cool waters near the thermocline of corridor lakes prior to arrival at spawning grounds, accumulated thermal units remain lower than if the animal remained in warmer river waters (Newell and Quinn, 2005; Roscoe *et al*., 2010). This tactic combined with changes in river temperature during migration produces a dynamic thermal experience that likely impacts physiological and disease-associated responses to fisheries capture. The Early Stuart population of sockeye salmon (*Oncorhynchus nerka*), for example, migrates ~1200 km from the mouth of the Fraser River to spawning grounds near the Stuart Lake system (Fig. 1); they begin this migration earlier than any other Fraser salmon population (median historical river entry date of 7 July) while the spring freshet is still diminishing and river temperatures are concurrently rising, and are thus faced with a narrow window of optimal migratory conditions (Macdonald *et al*., 2010; Reed *et al*., 2011). They also migrate at the same time as some spring Chinook salmon (*Oncorhynchus tshawytscha*) populations, which are the target of in-river First Nations gillnet fisheries. Declining abundance of returning adult Early Stuart sockeye salmon in recent decades has raised interest in how fishery-related bycatch mortality and river conditions may affect this population's continued viability.

To characterize the mechanisms contributing to post-release mortality and address fishery and managerial concerns regarding specific regulations, we conducted a long-term holding study using wild-caught Early Stuart sockeye salmon. This project was conducted in collaboration with First Nations user groups of the Lower Fraser Fisheries Alliance (LFFA) as well as managers and scientists of the Department of Fisheries and Oceans Canada (DFO). Concerns were raised among users and managers regarding the accuracy of the post-release mortality rate (60%) assigned by regulators to Early Stuart sockeye bycatch within the Chinook drift and set gillnet fishery that takes place during the Early Stuart sockeye migration. The primary purpose of our study was to test the variability of this post-release mortality estimate under different capture severities (i.e. entanglement durations) and a realistic thermal experience to inform fishery management and best practices of fishers. Furthermore, we sought to identify short-term effects of capture and predictive factors that distinguish fish that survive to the spawning period of Early Stuart sockeye from those that do not by using an array of physiological, environmental and disease-associated variables. Finally, we endeavoured to characterize relationships between infection intensities of potential pathogens at death with host physiology towards a mechanistic understanding of post-release mortality.

## Materials and Methods

The total migration duration from ocean departure to spawning grounds for Early Stuart sockeye salmon is ~3–4 weeks (Crossin *et al*., 2004; Macdonald *et al*., 2010; Reed *et al*., 2011). We captured individuals ~5 days into their upstream migration, ~150 river kilometers (rkm) from the mouth of the Fraser River in Yale, BC, using a 5.25-inch (13.3 cm) mesh gillnet (mesh size targeting Early Stuart sockeye). Fishing took place between 08:00 and 12:00 on 9 and 10 July 2013 and river temperature ranged between 16 and 17°C during collection. Fish were quickly and carefully removed from the net to minimize injury and stress and immediately placed into coolers filled with fresh river water. This type of capture was chosen as the most low-impact yet effective way of collecting Early Stuart sockeye; any observable impacts of collection (e.g. injury and lethargy) were factored into an overall condition score and incorporated into survival assessments (see below). A subset of fish was sacrificed river-side within 5 min of capture (*n* = 19) and sampled for gill tissue (2–3 filament tips, representing ~0.5 mg of tissue) and blood (~2 ml from the caudal vasculature; 21-gauge needle with lithium heparinized Vacutainer^®^, Becton-Dickson, NJ) to provide baseline data pertaining to condition at the time of capture (details on tissue storage and handling below). Live fish were placed in aerated truck-mounted tanks filled with cool (11–12°C), UV-treated and sand-filtered water for transport to the DFO Cultus Lake Salmon Research Lab at Cultus Lake, BC (40 min transit; Fig. 1). Fish were dip-netted from transport tanks and sequentially distributed among eight holding tanks (~8000 l; 16–17°C). Tank water at the facility was sourced from the neighbouring Cultus Lake, which was sand filtered, UV-treated, flow-through (e.g. not recirculated), and temperature controlled by manipulating the proportion of water from above or below the lake's thermocline. To achieve warmer temperatures, tanks were supplemented with boiler-heated shallow lake water. Tanks had a constant inflow above 30 l/min and were outfitted with a submersible pump creating a circular flow pattern around the tank periphery (~30 cm/s) which encouraged fish to slowly swim in place during holding.

Tanks were assigned to one of four treatments with two tank replicates per treatment group. The methods for this experiment were carried out under protocols approved by the Animal Care Committees of Fisheries and Oceans Canada (Pacific Region), the University of British Columbia (certificate A11-0215) and the University of Victoria (certificate 2012-030). Treatments included (i) a severe gillnet entanglement (20 min entanglement plus 1 min air exposure), (ii) a mild gillnet entanglement (20 s entanglement plus 1 min air exposure), (iii) a biopsied control without entanglement group and (iv) a control without biopsy or entanglement. Twenty-four to 48 h after collection, the standardized entanglement treatments were applied in the laboratory using an 8-inch (20.3 cm) mesh gillnet, which matches the mesh size used in the Fraser River Chinook fishery experiencing Early Stuart sockeye bycatch.

The gillnet treatment proceeded as follows: each fish was individually removed from its holding tank with a dip-net and immediately submerged in a treatment tank within the bag of the dip-net where the fish was then quickly entangled in the 8-inch mesh gillnet and then flipped out of the dip-net under water. This entanglement method was employed due to the large mesh size of the gillnet, which in our experience was too wide for Early Stuart sockeye to be caught *via* the gilled method. Our directive was not to quantify the causes of bycatch or encounter rates, but to understand effects of capture and release. Hence, this method was appropriate to achieve effective entanglement. After 20 min (severe) or 20 s (mild) of sustained entanglement, both the gillnet and fish were lifted out of the water and placed into a dip-net held in the air. After a 1-min air exposure, which included net removal and simulated a realistic time for bycatch landing and net removal, the fish was submerged in a flow-through, padded sampling trough for biopsy. Each fish was measured for fork length (FL; ±1 cm) and muscle lipid content (Fish Fatmeter Model-FM 692, Distell, Scotland, UK), biopsied for gill tissue (2–3 filament tips) and blood (~2 ml from the caudal vasculature; 21-gauge needle with lithium heparinized Vacutainer^®^, Becton-Dickson, NJ), externally tagged for identification (spaghetti-style tag, Floy^®^, WA), and then placed into a recovery tank. Its condition was recorded as an integer score from 0 to 6, which was a composite score of condition prior to experimental treatment [0 = no injury from the collection net and vigorous in the treatment net, 1 = mild abrasions (e.g. scale loss) but vigorous, 2 = moderately injured (e.g. skin loss) or lethargic, 3 = severely injured (e.g. bleeding or flesh loss) and lethargic] and condition following the experimental gillnet treatment (0–4, same criteria as above). Anesthetic was not used so as to mimic as much as possible the conditions of the fishery (see Cooke *et al*., 2005 for evaluation and validation of biopsy without anesthetic). Water temperature was constant throughout the treatment and sampling procedures (16–17°C; ≤2 min total time in trough). Biopsied control fish were similarly dip-netted from holding tanks, but bypassed gillnet and air exposure treatments to proceed directly to the sampling trough, henceforth following the biopsy protocol outlined above. Non-treatment air exposure associated with movement of biopsied control fish between tanks and the sampling trough was ≤10 s total. Non-biopsied control fish were not handled at all after collection. Experiment start for each individual corresponds to the time it entered the recovery tank; for non-biopsied control fish, start time corresponds to the earliest start time for treated fish. Due to a plumbing malfunction in their recovery tank, individuals from one holding tank (biopsied controls, *n* = 14) were excluded from long-term analyses. These individuals were, however, included in short-term analyses that did not relate to subsequent survival, but included condition and infection intensity at the time of the biopsy, prior to entering the recovery tank, relating to short-term effects of capture.

Tanks were checked at ≤4 h intervals between 08:00 and 24:00. Any individual displaying signs of morbidity (e.g. loss of equilibrium and surface gulping) was removed from the tank and euthanized; all fish surviving to the end of the spawning period, as determined by the duration of Early Stuart residence on spawning grounds and a sharp increase in (senescence-related) mortality of held fish, were euthanized. All euthanized fish were immediately biopsied as described above to preserve the integrity of RNA and blood properties. An adipose fin tissue sample was taken using a hole punch for DNA analysis. There was a gross examination of external and internal pathologies (e.g. *Saprolegnia* spp. fungus cover, organ abnormalities and lesions) while subsampling tissue from six additional major organs (liver, spleen, heart ventricle, head kidney and white muscle; brain alternated between RNA screening and histopathology) for microbe RNA screening and histopathology (histopathology data not shown).

Fish were held for the duration of their natural freshwater migration (~3 weeks) and spawning period (an additional 3 weeks that included staging, spawning and nest defence) to assess their survival during these periods associated with the experimental treatments. For the duration of the experiment, water temperature within all tanks was monitored and adjusted daily to mimic the thermal experience of a successful Early Stuart sockeye salmon that would be migrating towards spawning grounds in the same year as our study (Fig. 1). We constructed a thermal experience model in real time using thermal data loggers in place along the migration route (DFO Environmental Watch Program; http://www.pac.dfo-mpo.gc.ca/science/habitat/frw-rfo/index-eng.html) and migration rate estimates calculated for Early Stuart sockeye (Rand and Hinch, 1998). We estimated the geographic location of a successful migrant from the final date of collection until the end of the spawning period, including behavioural thermoregulation utilizing cool hypolimnetic water while passing through corridor lakes (Newell and Quinn, 2005; Mathes *et al*., 2010; Roscoe *et al*., 2010), and adjusted tank temperatures daily to match this estimated thermal experience. Briefly, for Early Stuart sockeye captured ~150 rkm from the mouth of the Fraser River, traveling at a ground speed of ~0.8m/s within the Fraser River mainstem (corresponding to ~4000 m^3^/s discharge) and then ~1 m/s in the Stuart and Nechako rivers, they would reach lake systems after ~10–11 days from the start of the study (between 21 and 23 July). To incorporate behavioural thermoregulation prior to arrival at spawning grounds, temperature was decreased to 11–12°C following simulated lake arrival on 24 July. Finally, on 28 July temperature was increased to 16°C and then lowered to 12°C to simulate movement out of the lakes, through the river, and on to cooler spawning grounds (Macdonald *et al*., 2012).

A subset of Early Stuart sockeye was sacrificed at spawning grounds near Takla Lake (*n* = 13; Fig. 1) and biopsied according to terminal sampling procedures described above to measure microbe prevalence on spawning grounds (7–8 August 2013).

### Laboratory analyses

Haematocrit and leucocrit values were measured immediately following blood sampling by calculating the volumes of red and white blood cell layers relative to total blood volume, respectively, after centrifugation (2 min at 10 000 *g*; LW Scientific^®^ ZIPocrit, GA, USA) in heparinized micro-capillary tubes (Drummond Scientific^®^, PA, USA). The remaining whole blood (~2 ml) was centrifuged within the Vacutainer^®^ for 7 min at 7000 *g* to remove plasma (Clay Adams Compact II centrifuge, NY, USA), which was then flash frozen in liquid nitrogen for subsequent analyses of hormones and metabolites. Gill tissue and other organ tissues were preserved in 1.5 ml of RNA*later*^®^ solution (Qiagen, MD, USA) for genomic analyses (whole brain in 3 ml). Percent lipid content of dorsal muscle was estimated using Fatmeter values and equations developed for sockeye (Crossin and Hinch, 2005). Stock identification as Early Stuart complex was confirmed *via* microsatellite DNA analysis of the adipose fin at the DFO Pacific Biological Station in Nanaimo, BC (Beacham *et al*., 2004). Plasma sodium, chloride, potassium, osmolality, lactate and glucose were measured using protocols described by Farrell *et al*. (2001); and cortisol, testosterone and oestradiol were examined using enzyme-linked immunosorbent assay (ELISA) kits (Neogen Corporation, KY, USA) following the manufacturer's protocols.

Genomic analyses were conducted at the DFO Pacific Biological Station using high-throughput nanofluidic qPCR (Fluidigm^®^ BioMark™ Dynamic Array, CA, USA) for quantification of relative RNA expression of disease-associated microbes and host stress- and immune-related biomarkers (Miller *et al*., 2014, 2016; Tables 1 and 2). Preserved tissue samples (~0.5 mg) were homogenized independently for 6–9 min in 600 µl TRI reagent (Ambion Inc., TX, USA) and 75 µl 1-bromo-3-chloropropane in microtubes using stainless steel beads and a MM301 mixer mill (Restch Inc., PA, USA). Whole brains were quartered and each section homogenized in 600 µl TRI reagent; 150 µl aliquots from each brain quarter were pooled into 600 µl of diluted brain homogenate prior to the addition of 1-bromo-3-chloropropane. Microtubes were then manually shaken for 1 min followed by 5 min at rest (repeated once), then centrifuged at 1500 *g* for 6.5 min. Aliquots of the aqueous phase (15 µl) from each tissue type were combined to produce a tissue pool from each individual fish. RNA was purified following the manufacturer's instructions using the ‘spin method’ for Magmax™-96 for Microarrays Kits (Albion Inc., TX, USA), with an additional DNase treatment to prevent DNA contamination. Extractions were performed using a Biomek FXP (Beckman-Coulter, ON, Canada) automated liquid handler. Quantity (*A*_260_) and quality (*A*_260_/*A*_280_ ratio) of purified RNA were examined *via* spectrophotometry. Total RNA in each sample was normalized (0.5 µg per sample for gill, 1.0 µg for pooled tissues) and cDNA was made using an Invitrogen™ SuperScript™ VILO™ (CA, USA) cDNA Synthesis Kit under PCR cycling conditions of 25°C for 10 min, 42°C for 60 min and 85°C for 5 min.

**Table 1:** Primer and probe sequences corresponding to stress and immunity biomarkers and three reference genes evaluated *via* qPCR on adult sockeye salmon (*Oncorhynchus nerka*).

Assay name	Gene information	Assay type	EST/Accession#	Primer and probe sequences	Efficiency	Source
B2M	Beta 2-microglobulin	Immune	AF180490	F—TTTACAGCGCGGTGGAGTC	0.92	Haugland *et al*. (2005)
				R—TGCCAGGGTTACGGCTGTAC		
				P—AAAGAATCTCCCCCCAAGGTGCAGG		
C3	Complement factor	Immune	U61753, AF271080	F—ATTGGCCTGTCCAAAACACA	0.93	Raida and Buchmann (2009)
				R—AGCTTCAGATCAAGGAAGAAGTTC		
				P—TGGAATCTGTGTGTCTGAACCCC		
CD4	Cell receptor	Immune	AY973028	F—CATTAGCCTGGGTGGTCAAT	0.83	Raida and Buchmann (2008)
				R—CCCTTTCTTTGACAGGGAGA		
				P—CAGAAGAGAGAGCTGGATGTCTCCG		
CD83	Cell receptor	Immune	AY263794	F—GATGCACCCCTTGAGAAGAA	0.76	Raida *et al*. (2011)
				R—GAACCCTGTCTCGACCAGTT		
				P—AATGTTGATTTACACTCTGGGGCCA		
Hep	Hepcidin	Immune	AF281354.1	F—GAGGAGGTTGGAAGCATTGA	0.82	Raida and Buchmann (2009)
				R—TGACGCTTGAACCTGAAATG		
				P—AGTCCAGTTGGGGAACATCAACAG		
IFNa	Interferon-α	Immune	AY216595	F—CGTCATCTGCAAAGATTGGA	0.78	Ingerslev *et al*. (2009)
				R—GGGCGTAGCTTCTGAAATGA		
				P—TGCAGCACAGATGTACTGATCATCCA		
IgMs	Immunoglobulin	Immune	S63348, AB044939	F—CTTGGCTTGTTGACGATGAG	0.79	Raida *et al*. (2011)
				R—GGCTAGTGGTGTTGAATTGG		
				P—TGGAGAGAACGAGCAGTTCAGCA		
IL-11	Cytokine	Immune	AJ535687	F—GCAATCTCTTGCCTCCACTC	0.79	Raida and Buchmann (2008)
				R—TTGTCACGTGCTCCAGTTTC		
				P—TCGCGGAGTGTGAAAGGCAGA		
IL-15	Cytokine	Immune	AJ555868.1	F—TTGGATTTTGCCCTAACTGC	0.82	Raida *et al*. (2011)
				R—CTGCGCTCCAATAAACGAAT		
				P—CGAACAACGCTGATGACAGGTTTTT		
IL-1R	Cytokine	Immune	AJ295296	F—ATCATCCTGTCAGCCCAGAG	0.80	Raida *et al*. (2011)
				R—TCTGGTGCAGTGGTAACTGG		
				P—TGCATCCCCTCTACACCCCAAA		
IRF1	Interferon regulatory factor 1	Immune	CB511515	F—CAAACCGCAAGAGTTCCTCATT	0.74	In house
				R—AGTTTGGTTGTGTTTTTGCATGTAG		
				P—CTGGCGCAGCAGATA		
MHCI	Major histocompatibility complex I	Immune		F—GCGACAGGTTTCTACCCCAGT	0.81	Ingerslev *et al*. (2009)
				R—TGTCAGGTGGGAGCTTTTCTG		
				P—TGGTGTCCTGGCAGAAAGACGG		
MHCII-B	Major histocompatibility complex IIβ	Immune	AF115533	F—TGCCATGCTGATGTGCAG	0.80	Raida and Buchmann (2008)
				R—GTCCCTCAGCCAGGTCACT		
				P—CGCCTATGACTTCTACCCCAAACAAAT		
MMP13	Matrix metalloproteinase	Immune	213514499	F—GCCAGCGGAGCAGGAA	0.81	Tadiso *et al*. (2011)
				R—AGTCACCTGGAGGCCAAAGA		
				P—TCAGCGAGATGCAAAG		
Mx	Antiviral protein	Immune		F—AGATGATGCTGCACCTCAAGTC	0.81	Eder *et al*. (2009)
				R—CTGCAGCTGGGAAGCAAAC		
				P—ATTCCCATGGTGATCCGCTACCTGG		
RIG-I	Retinoic acid inducible gene I	Immune	NM_001163699	F—ACAGCTGTTACACAGACGACATCA	0.81	Larsen *et al*. (2012)
				R—TTTAGGGTGAGGTTCTGTCCGA		
				P—TCGTGTTGGACCCCACTCTGTTCTCTC		
SHOP21	Salmon hyperosmotic protein 21	Immune	CA054269	F—GCGGTAGTGGAGTCAGTTGGA	0.76	In house
				R—GCTGCTGACGTCTCACATCAC		
				P—CCTGTTGATGCTCAAGG		
TF	Transferrin	Immune	D89083	F—TTCACTGCTGGAAAATGTGG	0.81	Raida and Buchmann (2009)
				R—GCTGCACTGAACTGCATCAT		
				P—TGGTCCCTGTCATGGTGGAGCA		
ATP5G3-C	ATP synthase	MRS	CB493164	F—GGAACGCCACCATGAGACA	0.79	In house
				R—CGCCATCCTGGGCTTTG		
				P—AGCCCCATTGCCTC		
C4B	Complement factor	MRS	CB518123	F—TCCAACCACATCGCATTATCC	0.73	In house
				R—ATCTCTGACACCACTGACCACAA		
				P—ATAGACAGGCTTCCC		
C7	Complement factor	MRS	CA052045	F—ACCTCTGTCCAGCTCTGTGTC	0.84	In house
				R—GATGCTGACCACATCAAACTGC		
				P—AACTACCAGACAGTGCTG		
EIF4E	Initiation factor	MRS	CA051191, CB496372	F—TCTGGAAACCCACACACAAAGA	1.00	In house
				R—GCGTTTTGAGGTTTGCATGTT		
				P—CCTGCCATAGCCACAC		
KCTD1	Potassium channel tetramerization domain	MRS	CA062065	F—TGTTTGTTAAAAGGGGACACAGTG	0.88	In house
				R—GTGAAGTGTTATCTGGGCTGAAAG		
				P—CTCCAAGGCTGAAAT		
MCSF	Macrophage colony stimulating factor	MRS	CA061415	F—GCTCTCTCAATCCTTGGCTTTAC	0.85	In house
				R—ACCAGCATAATTGAAAACCAGAGG		
				P—CTCAATGTCCTCAATGCT		
GR-2	Glucocorticoid receptor	Stress		F—TCCAGCAGCTATGCCAGTTCT	0.84	(Yada *et al*., 2007)
				R—TTGCCCTGGGTTGTACATGA		
				P—AAGCTTGGTGGTGGCGCTG		
HSC70	Heat shock cognate 70	Stress	CA052185	F—GGGTCACACAGAAGCCAAAAG	0.75	In house
				R—GCGCTCTATAGCGTTGATTGGT		
				P—AGACCAAGCCTAAACTA		
Hsp90	Heat shock protein 90	Stress	CB493960, CB503707	F—TGGGCTACATGGCTGCCAAG	0.80	In house
				R—TCCAAGGTGAACCCAGAGGAC		
				P—AGCACCTGGAGATCAA		
JUN	Transcription factor	Stress	CA056351	F—TTGTTGCTGGTGAGAAAACTCAGT	0.79	In house
				R—CCTGTTGCCCTATGAATTGTCTAGT		
				P—AGACTTGGGCTATTTAC		
78d16.1		Reference	CA056739	F—GTCAAGACTGGAGGCTCAGAG	0.84	In house
				R—GATCAAGCCCCAGAAGTGTTTG		
				P—AAGGTGATTCCCTCGCCGTCCGA		
COIL-P84-2		Reference	CA053789	F—GCTCATTTGAGGAGAAGGAGGATG	0.83	In house
				R—CTGGCGATGCTGTTCCTGAG		
				P—TTATCAAGCAGCAAGCC		
MRPL40		Reference	CK991258	F—CCCAGTATGAGGCACCTGAAGG	0.76	In house
				R—GTTAATGCTGCCACCCTCTCAC		
				P—ACAACAACATCACCA		

Assay type classifies genes by their association with immunity, stress or a mortality-related signature (MRS) predictive of migration failure of wild salmon (Miller *et al*., 2011). References and average qPCR efficiencies are provided; in-house designs were conducted by the Molecular Genetics Laboratory at the Pacific Biological Station, Nanaimo, BC.

**Table 2:** Abbreviations, names and types of microbes suspected or known to cause disease in Pacific salmon in British Columbia, Canada, evaluated *via* qPCR on adult sockeye salmon (*Oncorhynchus nerka*)

Assay abbreviation	Microbe full name	Type	Prevalence *en route* and held	Prevalence at spawning grounds	Primer and probe sequences	Efficiency	Reference
ae_hyd	*Aeromonas hydrophila*	Bacterium	1	15	F—ACCGCTGCTCATTACTCTGATG	0.91	Lee *et al*. (2006)
					R—CCAACCCAGACGGGAAGAA		
					P—TGATGGTGAGCTGGTTG		
ae_sal	*Aeromonas salmonicida*	Bacterium	0	0	F—TAAAGCACTGTCTGTTACC	0.96	Keeling *et al*. (2013)
					R—GCTACTTCACCCTGATTGG		
					P—ACATCAGCAGGCTTCAGAGTCACTG		
re_sal	*Renibacterium salmoninarum*	Bacterium	0	0	F—CAACAGGGTGGTTATTCTGCTTTC	0.93	Powell *et al*. (2005)
					R—CTATAAGAGCCACCAGCTGCAA		
					P—CTCCAGCGCCGCAGGAGGAC		
c_b_cys	*Candidatus Branchiomonas cysticola*	Bacterium	96	100	F—AATACATCGGAACGTGTCTAGTG	0.90	Mitchell *et al*. (2013)
					R—GCCATCAGCCGCTCATGTG		
					P—CTCGGTCCCAGGCTTTCCTCTCCCA		
ye_ruc	*Yersinia ruckeri*	Bacterium	0	0	F—TGCCGCGTGTGTGAAGAA	0.93	Glenn *et al*. (2011)
					R—ACGGAGTTAGCCGGTGCTT		
					P—AATAGCACTGAACATTGAC		
fl_psy	*Flavobacterium psychrophilum*	Bacterium	55	100	F—GATCCTTATTCTCACAGTACCGTCAA	0.80	Duesund *et al*. (2010)
					R—TGTAAACTGCTTTTGCACAGGAA		
					P—AAACACTCGGTCGTGACC		
pch_sal	*Piscichlamydia salmonis*	Bacterium	0	0	F—TCACCCCCAGGCTGCTT	0.87	Nylund *et al*. (2008)
					R—GAATTCCATTTCCCCCTCTTG		
					P—CAAAACTGCTAGACTAGAGT		
pisck_sal	*Piscirickettsia salmonis*	Bacterium	0	0	F—TCTGGGAAGTGTGGCGATAGA	0.95	Corbeil *et al*. (2003)
					R—TCCCGACCTACTCTTGTTTCATC		
					P—TGATAGCCCCGTACACGAAACGGCATA		
rlo	*Rickettsia-like organism*	Bacterium	78	69	F—GGCTCAACCCAAGAACTGCTT	0.89	Lloyd *et al*. (2011)
					R—GTGCAACAGCGTCAGTGACT		
					P—CCCAGATAACCGCCTTCGCCTCCG		
sch	*Gill chlamydia*	Bacterium	0	0	F—GGGTAGCCCGATATCTTCAAAGT	0.95	Duesund *et al*. (2010)
					R—CCCATGAGCCGCTCTCTCT		
					P—TCCTTCGGGACCTTAC		
vi_ang	*Vibrio anguillarum*	Bacterium	0	0	F—CCGTCATGCTATCTAGAGATGTATTTGA	0.96	In house
					R—CCATACGCAGCCAAAAATCA		
					P—TCATTTCGACGAGCGTCTTGTTCAGC		
vi_sal	*Vibrio salmonicida*	Bacterium	0	0	F—GTGTGATGACCGTTCCATATTT	0.91	In house
					R—GCTATTGTCATCACTCTGTTTCTT		
					P—TCGCTTCATGTTGTGTAATTAGGAGCGA		
aspv	Atlantic salmon paramyxovirus	Virus	0	0	F—CCCATATTAGCAAATGAGCTCTATCTT	0.92	Nylund *et al*. (2008)
					R—CGTTAAGGAACTCATCATTGAGCTT		
					P—AGCCCTTTTGTTCTGC		
pmcv	Piscine totivirus (CMS)	Virus	4	15	F—TTCCAAACAATTCGAGAAGCG	0.92	Løvoll *et al*. (2010)
					R—ACCTGCCATTTTCCCCTCTT		
					P—CCGGGTAAAGTATTTGCGTC		
ver	Viral encephalopathy and retinopathy virus	Virus	0	0	F—TTCCAGCGATACGCTGTTGA	1.02	Korsnes *et al*. (2005)
					R—CACCGCCCGTGTTTGC		
					P—AAATTCAGCCAATGTGCCCC		
vhsv	Viral haemorrhagic septicaemia virus	Virus	0	0	F—ATGAGGCAGGTGTCGGAGG	0.86	Garver *et al*. (2011)
					R—TGTAGTAGGACTCTCCCAGCATCC		
					P—TACGCCATCATGATGAGT		
omv	Salmonid herpesvirus	Virus	0	0	F—GCCTGGACCACAATCTCAATG	0.95	In house
					R—CGAGACAGTGTGGCAAGACAAC		
					P—CCAACAGGATGGTCATTA		
sav	Salmon alphavirus	Virus	0	0	F—CCGGCCCTGAACCAGTT	0.99	Andersen *et al*. (2007)
					R—GTAGCCAAGTGGGAGAAAGCT		
					P—TCGAAGTGGTGGCCAG		
ven	Viral erythrocytic necrosis virus	Virus	0	0	F—CGTAGGGCCCCAATAGTTTCT	0.96	James Winton, pers. comm.
					R—GGAGGAAATGCAGACAAGATTTG		
					P—TCTTGCCGTTATTTCCAGCACCCG		
pspv	Pacific salmon parvovirus	Virus	0	0	F—CCCTCAGGCTCCGATTTTTAT	NA	In house
					R—CGAAGACAACATGGAGGTGACA		
					P—CAATTGGAGGCAACTGTA		
prv	Piscine reovirus (HSMI, CMS)	Virus	0	0	F—TGCTAACACTCCAGGAGTCATTG	0.85	Wiik-Nielsen *et al*. (2012)
					R—TGAATCCGCTGCAGATGAGTA		
					P—CGCCGGTAGCTCT		
ihnv	Infectious haematopoietic necrosis virus	Virus	0	0	F—AGAGCCAAGGCACTGTGCG	0.87	Purcell *et al*. (2013)
					R—TTCTTTGCGGCTTGGTTGA		
					P—TGAGACTGAGCGGGACA		
cr_sal	*Cryptobia salmositica*	Parasite	0	15	F—TCAGTGCCTTTCAGGACATC	0.89	In house
					R—GAGGCATCCACTCCAATAGAC		
					P—AGGAGGACATGGCAGCCTTTGTAT		
ce_sha	*Ceratonova shasta*	Parasite	96	85	F—CCAGCTTGAGATTAGCTCGGTAA	0.93	Hallett and Bartholomew (2006)
	(formerly *Ceratomyxa shasta*)				R—CCCCGGAACCCGAAAG		
					P—CGAGCCAAGTTGGTCTCTCCGTGAAAAC		
de_sal	*Dermocystidium salmonis*	Parasite	1	0	F—CAGCCAATCCTTTCGCTTCT	0.90	In house
					R—GACGGACGCACACCACAGT		
					P—AAGCGGCGTGTGCC		
fa_mar	*Facilispora margolisi*	Parasite	1	0	F—AGGAAGGAGCACGCAAGAAC	0.92	In house
					R—CGCGTGCAGCCCAGTAC		
					P—TCAGTGATGCCCTCAGA		
gy_sal	*Gyrodactylus salaris*	Parasite	0	0	F—CGATCGTCACTCGGAATCG	0.89	Collins *et al*. (2010)
					R—GGTGGCGCACCTATTCTACA		
					P—TCTTATTAACCAGTTCTGC		
ic_mul	*Ichthyophthirius multifiliis*	Parasite	83	100	F—AAATGGGCATACGTTTGCAAA	0.9	In house
					R—AACCTGCCTGAAACACTCTAATTTTT		
					P—ACTCGGCCTTCACTGGTTCGACTTGG		
ku_thy	*Kudoa thyrsites*	Parasite	10	23	F—TGGCGGCCAAATCTAGGTT	0.91	Funk *et al*. (2007)
					R—GACCGCACACAAGAAGTTAATCC		
					P—TATCGCGAGAGCCGC		
lo_sal	*Loma salmonae*	Parasite	79	77	F—GGAGTCGCAGCGAAGATAGC	0.93	In house
					R—CTTTTCCTCCCTTTACTCATATGCTT		
					P—TGCCTGAAATCACGAGAGTGAGACTACCC		
my_arc	*Myxobolus arcticus*	Parasite	18	23	F—TGGTAGATACTGAATATCCGGGTTT	0.89	In house
					R—AACTGCGCGGTCAAAGTTG		
					P—CGTTGATTGTGAGGTTGG		
my_ins	*Myxobolus insidiosus*	Parasite	0	0	F—CCAATTTGGGAGCGTCAAA	0.83	In house
					R—CGATCGGCAAAGTTATCTAGATTCA		
					P—CTCTCAAGGCATTTAT		
my_cer	*Myxobolus cerebralis*	Parasite	0	0	F—GCCATTGAATTTGACTTTGGATTA	0.99	Kelley *et al*. (2004)
					R—ACCATTCATGTAAGCCCGAACT		
					P—TCGAAGCCTTGACCATCTTTTGGCC		
ne_per	*Neoparamoeba perurans*	Parasite	0	0	F—GTTCTTTCGGGAGCTGGGAG	1.05	Fringuelli *et al*. (2012)
					R—GAACTATCGCCGGCACAAAAG		
					P—CAATGCCATTCTTTTCGGA		
nu_sal	*Nucleospora salmonis*	Parasite	0	0	F—GCCGCAGATCATTACTAAAAACCT	0.94	Foltz *et al*. (2009)
					R—CGATCGCCGCATCTAAACA		
					P—CCCCGCGCATCCAGAAATACGC		
pa_ther	*Paranucleospora theridion*	Parasite	13	23	F—CGGACAGGGAGCATGGTATAG	0.92	Nylund *et al*. (2010)
					R—GGTCCAGGTTGGGTCTTGAG		
					P—TTGGCGAAGAATGAAA		
pa_pse	*Parvicapsula pseudobranchicola*	Parasite	0	0	F—CAGCTCCAGTAGTGTATTTCA	0.95	Jørgensen *et al*. (2011)
					R—TTGAGCACTCTGCTTTATTCAA		
					P—CGTATTGCTGTCTTTGACATGCAGT		
pa_kab	*Parvicapsula kabatai*	Parasite	2	8	F—GTCGGATGATAAGTGCATCTGATT	0.97	In house
					R—ACACCACAACTCTGCCTTCCA		
					P—TGCGACCATCTGCACGGTACTGC		
te_bry	*Tetracapsuloides bryosalmonae*	Parasite	4	85	F—GCGAGATTTGTTGCATTTAAAAAG	0.89	Bettge *et al*. (2009)
					R—GCACATGCAGTGTCCAATCG		
					P—CAAAATTGTGGAACCGTCCGACTACGA		
pa_min	*Parvicapsula minibicornis*	Parasite	100	100	F—AATAGTTGTTTGTCGTGCACTCTGT	0.88	Hallett and Bartholomew (2009)
					R—CCGATAGGCTATCCAGTACCTAGTAAG		
					P—TGTCCACCTAGTAAGGC		
sp_des	*Sphaerothecum destruens*	Parasite	1	23	F—GCCGCGAGGTGTTTGC	0.89	In house
					R—CTCGACGCACACTCAATTAAGC		
					P—CGAGGGTATCCTTCCTCTCGAAATTGGC		
sp_sal	*Spironucleus salmonicida*	Parasite	0	0	F—AACCGGTTATTCGTGGGAAAG	0.91	In house
					R—TTAACTGCAGCAACACAATAGAATACTC		
					P—TGCCAGCAGCCGCGGTAATTC		
ic_hof	*Ichthyophonus hoferi*	Parasite	0	0	F—GTCTGTACTGGTACGGCAGTTTC	0.91	White *et al*. (2013)
					R—TCCCGAACTCAGTAGACACTCAA		
					P—TAAGAGCACCCACTGCCTTCGAGAAGA		
na_sal	*Nanophyetus salmincola*	Fluke	0	0	F—CGATCTGCATTTGGTTCTGTAACA	0.88	In house
					R—CCAACGCCACAATGATAGCTATAC		
					P—TGAGGCGTGTTTTATG		

Prevalence values describe percent positive detections among Early Stuart sockeye collected in the Fraser River at Yale, BC (*n* = 107; includes individuals sacrificed river-side at collection and those held for up to 40 days) and among those sacrificed at spawning grounds (*n* = 13; near Takla Lake, 7–8 August 2013). Primer and probe sequences with references and qPCR efficiencies are provided; in-house designs were conducted by the Molecular Genetics Laboratory at the Pacific Biological Station, Nanaimo, BC.

Given the nanolitre volumes of substrate incorporated into each qPCR chamber of the Biomark™, samples must first undergo a pre-amplification step consisting of a multiplex PCR including all target assay primers to achieve high sensitivity detections (for more information, see Miller *et al*., 2016). Following the manufacturer's protocols, a mix of forward and reverse primers corresponding to all targeted microbe and host biomarkers (200 nM primer mix; 1.3 µl total volume) was combined with 2.5 µl TaqMan^®^PreAmp Master Mix (Applied Biosystems, CA, USA and added to 1.3 µl cDNA; PCR cycling commenced at 95°C for 10 min followed by 15 cycles of 95°C for 10 s and 60°C for 4 min. Any remaining nucleotides and primers were removed using ExoSAP-IT^®^ PCR Product Cleanup (MJS BioLynx Inc., ON, Canada) cycled at 37°C for 15 min then 80°C for 15 min. Each sample was then diluted 5-fold with suspension buffer (TEKnova, CA, USA) so as not to overwhelm the subsequent qPCR analysis. Controls were incorporated among samples in duplicate during the extraction, pre-amplification and qPCR steps, including both positive (pooled cDNA samples from multiple individuals) and negative controls (suspension buffer); serial dilutions of pre-amplified pooled host samples and synthetic microbe sequence clones were also included among samples on each dynamic array during the final qPCR (Miller *et al*., 2016).

Biomarker and microbe assays were run in duplicate and included three reference genes to ensure viability of samples (i.e. routine host gene expression). Sample (TaqMan^®^ Gene Expression Master Mix, GE Sample Loading Reagent and pre-amplified cDNA) and assay (primer pair [9 µM], probe [2 µM], Assay Loading Reagent) mixes were individually plumbed into single reaction chambers using integrated fluidic circuits of the IFC controller prior to the qPCR cycling. The qPCR thermal cycling profile followed the GE 96 X 96 Standard v1.pcl. (TaqMan^®^) protocol. Passive reference dye was used to confirm that all 9216 wells contained substrate. Two probes were measured in each reaction chamber: one pertained to the target amplicon (FAM) and the other to microbe clone controls (VIC). Any sample reaction chamber found to be VIC positive was removed as suspected clone contamination. Cycle threshold (Ct) replicates were averaged for all samples; in the case of failed replicates, host biomarkers were assigned the single positive value, but any microbe not positive for both replicates was designated a negative detection. Host genes were normalized to the average of the three reference genes and relative expression was calculated using the 2^−ΔΔCt^ method (Livak and Schmittgen, 2001). Predetermined total copy numbers of synthetic microbe clone dilutions were used to create a standard curve to back calculate RNA copy numbers of microbes from Ct values measured in samples. Throughout the analyses herein, biomarker results are represented as relative expression and microbe infection intensity (RNA copy number) referred to as ‘productivity’.

We measured microbe productivities *via* the RNA expression of each microbe. Because primers and probes were designed to different gene types with varying functions (e.g. ribosomal 16S and surface array) depending on the target species, our conclusions are limited to describing variation among hosts within each microbe species. Comparisons among species would be misleading because different target genes are expressed at different rates. We chose RNA rather than DNA quantification so as to include RNA viruses in our screening approach and to represent changes in active expression of living microbes as opposed to direct quantification of potentially inactive DNA. Microbe productivity as we define it here is thus a measure of the relative activity of each microbe.

### Statistical analyses

Survival analysis was used to identify differences in survival among treatment groups and sexes to the peak of the spawning period for this population (20 days post-treatment; dpt) following treatment using the *survdiff* and *coxph* functions in the *survival* library in Program R (Therneau, 2014; R Core Team, 2015). Assumptions of the model, including proportional hazards, influential observations and linearity, were evaluated. Survival (>20 dpt) was also examined by treatment and sex using generalized linear models (GLMs) with a binomial response. GLMs were constructed including and excluding non-biopsied controls; this approach allowed us to examine the effect of the biopsy alone on survival and to identify survival differences between gillnetted fish and controls with and without the additional handling associated with the biopsy.

Short-term effects of capture on host physiology and microbe productivity were assessed by comparing samples taken from fish sacrificed river-side at the time of collection (immediately following capture, T0; *n* = 19) with non-lethal biopsy samples taken 1–2 days after fish collection (biopsied control group, T1; *n* = 28). Blood plasma indices of maturity, stress and osmoregulatory impairment at T0 and T1 were log-transformed if necessary to meet assumptions of normality. GLMs were constructed, with time and sex as predictor variables including an interaction term, and each physiological variable as the response. Principal component (PC) analysis was used to identify and characterize shifts in gene expression patterns (28 biomarkers of stress and immunity; see Table 1) measured in gill at T0 (*n* = 20) and at T1 (*n* = 22). Analysis of variance (ANOVA) was used to determine if the position of individuals along PC axes was correlated with sex or sampling date (T0, T1), and included an interaction term. Short-term changes in microbe productivity in gill were identified using hurdle models with a negative binomial response distribution; this approach conducts step-wise tests for differences in the presence of zeros (i.e. changes in prevalence between time points) and continuous positive values (i.e. microbe productivity as estimated by RNA copy number in positive detections). Microbe copy numbers were log-transformed prior to all analyses. We examined the effect of microbe richness on survival to 20 dpt of gillnet treated and biopsied control fish (*n* = 61) using a GLM with sex and treatment as cofactors.

We used a non-parametric multivariate classification tree model to identify physiological and environmental factors associated with survival to the spawning period (20 dpt) using the *rpart* library and *cartware* functions in Program R (De'ath and Fabricius, 2000; De'ath, 2002; Compton, 2006; Therneau *et al*., 2015). This analysis was restricted to fish that were exposed to gillnet treatments, including both severe and mild entanglements (*n* = 51), therefore including a mix of exposure times relevant to the fishery. The technique uses recursive partitioning to identify distinguishing variables among pre-defined groups (i.e. success or failure to survive to the spawning period). Simply, the analysis identifies the variable with the greatest power to distinguish between predefined groups, repeating this partitioning at each ‘branch’ until terminal nodes (partitioned collections of individuals at branch tips) reach sufficient correct classification. Fifty-two variables were included in the initial partitioning (Table 3), which when applied for descriptive purposes can handle large variable to sample ratios. The classification tree model was constructed using the ‘gini’ index as the splitting criteria, prior probability of group assignment was proportional to group sample sizes at each partition, and further partitioning was stopped within one standard error of the minimum relative error. Primary and surrogate splits were examined as well as variable importance regardless of incorporation in the final tree. The effectiveness of the model was examined using the Kappa chance-corrected error reduction rate. Model significance was assessed using Monte Carlo resampling with 100 random permutations of the grouping variable (success) with the derived tree size (three leaves) and variables (see Results section) of the final model; if *P* < 0.05, the correct classification rate of the original model was deemed sufficiently high relative to the distribution from random trees.

**Table 3:** Variables included in the multivariate classification tree analysis using survival to the spawning period of Early Stuart sockeye (>20 days post-treatment) as the grouping factor

Type	Variables
Environmental/morphological	Gillnet exposure time, sex, total condition score, length, stock
Microbes	c_b_cys, ce_sha, fl_psy, ic_mul, lo_sal, pa_min, rlo
Gene expression biomarkers of stress and immunity	ATP5G3C, B2M, C3, C4B, C7, CD4, CD83, EIF4E, GR2, hep, HSC70, Hsp90, IFNa, IgMs, IL11, IL15, IL1R, IRF1, JUN, KCTD1, MCSF, MHCI, MHCIIB, MMP13, Mx, RIGI, SHOP21, TF
Clinical variables (hormones, metabolites, ions and other physiological indicators)	Chloride, osmolality, sodium, potassium, muscle lipid, cortisol, oestradiol, testosterone, glucose, lactate, haematocrit, leucocrit

Full microbe names can be found in Table 2.

Because the purpose of our study was primarily focussed on the impact of capture severity on survival, we allowed individuals to progress to the stage of morbidity prior to sacrifice and re-sampling, rather than sacrificing all individuals simultaneously. Therefore, mortality took place over an extended temporal period (weeks–months) and was furthermore confounded with treatment and sex (see Survival section). Samples that were taken at death (i.e. terminal variables) are hence subject to an unknown relative influence of senescence or maturation trajectories, gillnet treatments and sex. Comparison of terminal variables among treatment groups is therefore fraught with speculation given this temporal confounding. We therefore limit our analysis of terminal variables to characterize trends in microbe productivities with time and qualitatively describing relationships among terminal microbe productivities and physiological variables.

To test the assumption that greater microbe productivities would be apparent in fish that die prematurely (as a proxy for advanced infection states), we used logistic and linear regression of days surviving with microbe prevalence and productivity, respectively. Linear regressions were limited to positive microbe detections with adequate sample sizes. A negative slope (*P* < 0.05) was assumed to represent higher microbe productivity in premature mortalities, suggesting potential pathogenicity, whereas a positive or zero slope would demonstrate lower or no difference in productivity in premature mortalities relative to fish that survived to the spawning period. The latter scenarios suggest no impact of microbe productivity on the host, or possibly a decreased threshold for microbe productivity tolerated by the host. We used microbe productivity values derived from pooled tissues rather than from the gill alone for a more comprehensive representation of microbes across tissues. Relationships between microbe productivities were evaluated using Spearman's Rank Correlation. This analysis was conducted using pooled tissue data for microbes with adequate sample sizes to obtain reliable results (see Table 2 for prevalence information). Spearman's correlation coefficients were calculated for all complete pairs, where both observations were positive detections, between *Candidatus* Branchiomonas cysticola (*n* = 50), *Ceratonova shasta* (*n* = 54), *Flavobacterium psychrophilum* (*n* = 42), *Ichthyophthirius multifiliis* (*n* = 63), *Loma salmonae* (*n* = 57), *Parvicapsula minibicornis* (*n* = 82) and Rickettsia-like organism (RLO; *n* = 56). Agreement between gill and pooled tissue microbe detections was assessed by presence–absence (percent agreement) and by productivity using linear regression on positive detections with a Breusch–Pagan test for heteroscedasticity (data from individuals sampled at death; *n* = 83).

Relationships between microbe productivities and host biomarkers of stress and immunity were characterized using Kruskal's non-metric multidimensional scaling (NMDS) in concert with the *envfit* function for fitting extrinsic variables in the *vegan* library in Program R (Oksanen *et al*., 2016). NMDS is a robust unconstrained ordination method (e.g. Hülber *et al*., 2009) that reduces the dimensionality of community data sets and establishes relationships among samples based on their composition (Minchin, 1987). We used the *metaMDS* function to create a Bray–Curtis distance matrix of individual fish based on their microbe communities (e.g. productivities of all microbe species measured in pooled tissues at death, *n* = 42), and then characterized their relationships with host biomarkers of stress and immunity as well as days surviving, treatment and sex. Prior to the analysis, microbe RNA copy numbers were transformed to a proportion of the total copies of each microbe species across all samples (i.e. column standardized), then expressed as a proportion of the total normalized values for each individual fish across microbe species (i.e. row standardized). The analysis was restricted to microbe species with greater than 10 positive detections to avoid a bias towards rare species. Two dimensions were included in the ordination, which was determined as the fewest possible axes with sufficient agreement between calculated and plotted distances (i.e. low stress). Species (microbe) scores were calculated as weighted averages in the two-dimensional space. A Monte–Carlo permutation test was used to determine the significance of the ordination (McCune *et al*., 2002; McGarigal, 2015). Genomic, clinical (blood properties, muscle lipid) and environmental (treatment, sex, days surviving) variables were fit onto the ordination using the *envfit* function, which maximizes the correlation between projected points and fitted variables. Resulting vectors represent the direction and relative strength (vector length) of the correlation; however, vector lengths for clinical variables were shortened to improve readability of the final plot. Variable goodness of fit (*r*^2^) and ‘significance’ (*P*) were assessed using permutation of environmental variables; a cutoff of *P* < 0.10 was applied for inclusion of external variables in the final descriptive diagram as demonstrating sufficient change along the ordination gradient to reliably enhance our understanding of host responses associated with microbe community structure.

## Results

### Survival

**Figure 2:**
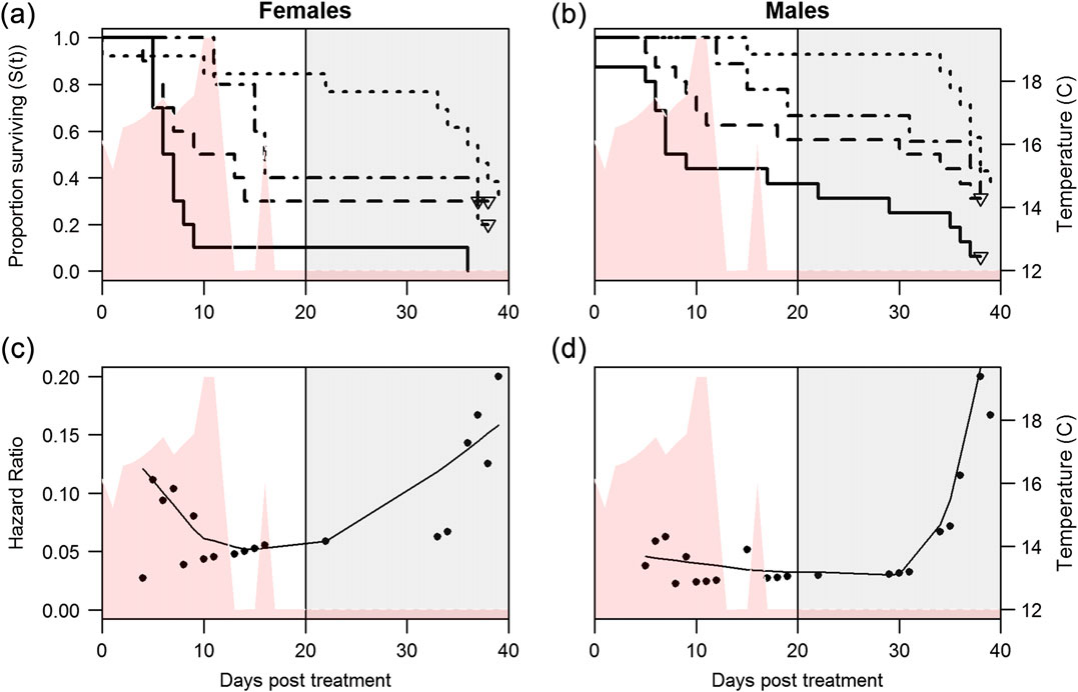
Kaplan–Meier survival curves are shown for female (**a**) and male (**b**) Early Stuart sockeye exposed to severe gillnet entanglement (20 min plus 1 min air exposure, *n* = 26; solid), mild gillnet entanglement (20 s plus 1 min air, *n* = 26; dashed), biopsied controls (*n* = 14; dot-dashed) and non-biopsied controls (*n* = 27; dotted). Triangles are censored data points. The grey shaded area corresponds to the spawning period of this population including nest defence. The red shaded area shows the temperature (°C) of all holding tanks through course of the study, which follows the modelled thermal experience of an Early Stuart sockeye migrant in the Fraser River in 2014. Daily hazard ratios for females (**c**) and males (**d**) are plotted as a function of time (all treatments combined) with a solid line lowess smoothing function. Hazard ratios correspond to the number of mortalities divided by the total survivors on each day the mortality occurred.

Based on the comparison of the control fish that were not handled at all following collection and the control fish that were biopsied, the biopsy itself had significant effects on percent mortality before the spawning period (Fig. 2, Table 4). The low sample size of biopsied controls relative to other control and treatment groups, however, warrants caution in interpreting the survival estimates for biopsied controls. Among biopsied controls, 33% of males and 60% of females died before the spawning period, while only 7% of male and 15% of female non-biopsied controls died before the spawning period. Percent mortality further increased with both entanglement intensities: severe entanglement resulted in the greatest mortality (males: 62%, females: 90%), followed by mild entanglement (males: 44%, females: 70%). Mortality of gillnet-treated fish did not occur until 5–10 days after entanglement, depending on entanglement severity and sex, and mortality among biopsied controls was delayed by 10–15 days after the biopsy. After accounting for mortality due to capture, holding, and biopsy sampling, we can expect ~10–55% of females exposed to mild entanglement to die before the spawning period, increasing to 30–75% following severe entanglement, while males would be expected to show 11–37% mortality before the spawning period following mild entanglement and 29–55% mortality following severe entanglement. The minimum of each mortality range was calculated using the percent mortality of biopsied controls and the maximum using non-biopsied controls. Estimates are presented as a range of values to account for uncertainty regarding the effect of non-lethal biopsy sampling, which could be additive or masking in its impact on survival.

**Table 4:** Sample sizes (*n*), mean days surviving (±standard error) and percent mortality prior to the spawning period (20 days post-treatment) for female (F) and male (M) Early Stuart sockeye salmon by treatment

	*n*		Days surviving		Mortality prior to spawning period	
	F	M	F	M	F (%)	M (%)
Severe gillnet	10	16	9.4 ± 1.8	16.3 ± 2.8	90	62
Mild gillnet	10	16	17.0 ± 2.8	24.4 ± 2.7	70	44
Biopsied control	5	9	23.4 ± 3.5	29.6 ± 2.9	60	33
Control	13	14	31.2 ± 2.4	36.0 ± 1.2	15	7
Total	38	55	20.7 ± 1.5	25.9 ± 1.4	55	38

Evaluation of the assumptions of the survival analysis revealed that the proportional hazards assumption was violated for gillnet-treated fish (*P*-values < 0.020), wherein the risk of death due to gillnet entanglement was high in the first 10 days and then decreased. Although this information is relevant to the study, it prohibited application of the survival analysis in identifying differences in survival among treatment groups. By stratifying treatment groups, the effect of sex on survival could be evaluated (within treatments) and was found to have a significant effect (*χ*^2^ = 6.9, *P* = 0.009), with males experiencing less than half (43%, *P* = 0.010) of the daily mortality risk that females experienced (model concordance = 0.611, *r*^2^ = 0.069, Likelihood ratio test *P* = 0.011). GLMs used to identify differences in survival to the spawning period showed higher mortality among biopsied controls (*P* = 0.010), 20-s gillnet (*P* = 0.001) and 20-min gillnet groups (*P* < 0.001) relative to non-biopsied controls (odds ratios = 11.6, 18.1, and 38.2, respectively), and lower mortality of males relative to females (odds ratio = 0.3, *P* = 0.029). Compared with biopsied controls, however, gillnet treatments did not significantly increase mortality (*P*-values > 0.10), though sex-specific differences were again significant (male odds ratio = 0.3, *P* = 0.023). Mortality and subsequent increases in total hazard ratios after 30 dpt (Fig. 2) are attributable to senescence.

### Short-term effects of capture

**Figure 3:**
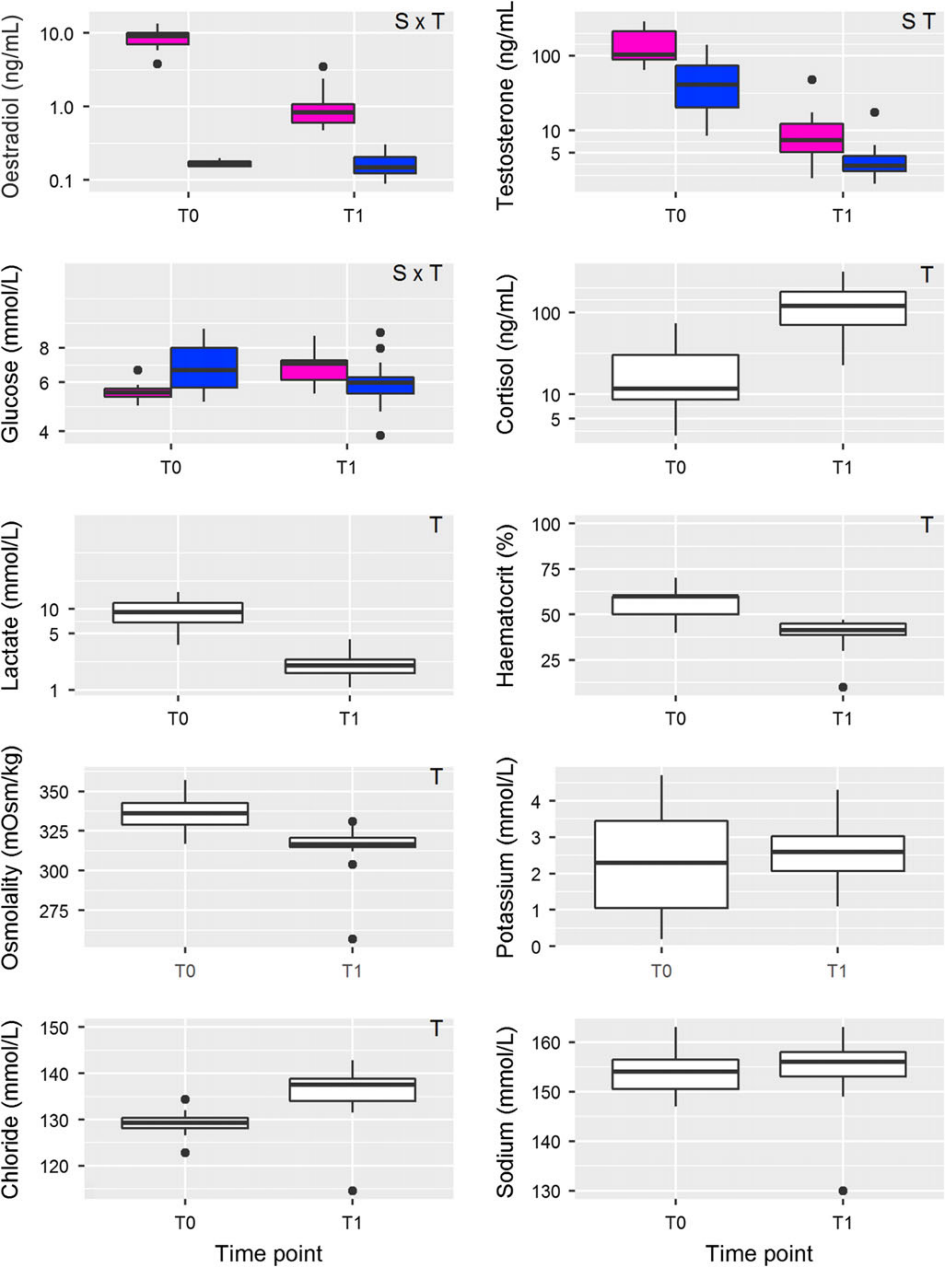
Box plots illustrating blood plasma indices of maturation (oestradiol and testosterone), metabolic stress (glucose, cortisol, lactate, haematocrit), and osmoregulatory and ionic imbalance (osmolality, potassium, chloride, sodium) measured in Early Stuart sockeye at the time of gillnet capture (T0; *n* = 19) and 2 days following gillnet capture (T1; *n* = 28). Oestradiol, testosterone, cortisol and glucose models included a significant sex factor showing differential changes for females (pink) and males (blue) at each time point. Letters at the top right of each plot denote significant differences (*P* < 0.05) between timepoints (T), sexes (S) or an interaction between the terms (S × T).

Plasma lactate, osmolality and haematocrit were significantly lower in fish sampled 1–2 days following capture (T1) relative to individuals sampled immediately after gillnet capture in the river (T0, *P*-values < 0.001), while cortisol and chloride were higher at T1 relative to T0 (*P*-values < 0.001; Fig. 3) with no sex-specific differences. Sex hormones (oestradiol and testosterone) were lower in males relative to females (*P*-values < 0.001), and both were reduced at T1 in males and females (*P*-values < 0.001), with a more dramatic decrease in oestradiol in females compared with males (interaction *P* < 0.001). Glucose levels differed between males and females (*P* = 0.013), and were elevated in females and depressed in males at T1 relative to T0 (sampling date: *P* = 0.012; interaction: *P* = 0.003).

**Figure 4: (a):**
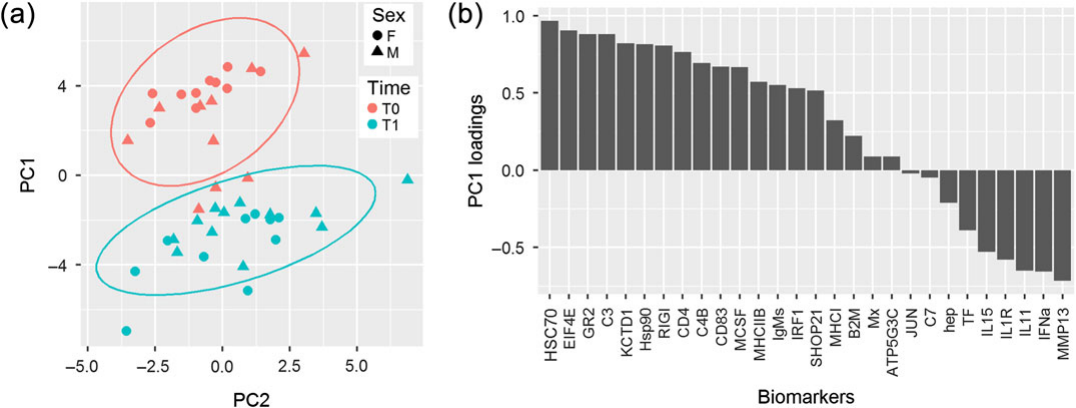
PC analysis of gene expression in gill tissue (28 biomarkers of stress and immunity) at the time of gillnet capture (T0; orange) and 2 days following capture (T1; blue). Ellipses represent 95% confidence intervals for each group cluster. (**b**) PC loadings of genomic biomarker variables.

Gene expression of targeted stress and immune biomarkers in the gill differed between fish sampled at T0 and at T1 (Fig. 4). PC1 explained much of the total variance (38%), and PC2 an additional 15%; a Monte–Carlo randomization test identified the first two components as significant (*P* < 0.001), though only the first component showed significant associations with sampling date (ANOVA: *P* < 0.001) and interaction between date and sex (*P* = 0.003), but no main effect of sex (*P* = 0.671). Individuals sampled at T0 loaded positively on PC1, while those sampled at T1 loaded negatively. Most of the biomarkers loaded positively on PC1, suggesting enhanced positive regulation of these genes at the time of capture relative to the days following. Sex-specific differences were noted among T0 fish, but not among T1 fish, where females clustered closely and positively on PC1 unlike males that had a greater range in their positions along PC1. Positively loaded biomarkers included many aspects of the stress response such as GR2, HSP90 and SHOP21 (loadings = 0.88, 0.82, and 0.51, respectively; Iwama *et al*., 1998; Pan *et al*., 2002; Yada *et al*., 2007) and several aspects of immunity such as HSC70, C3, RIG1 and CD4 (loadings = 0.97, 0.88, 0.81, and 0.77, respectively). Negatively loaded biomarkers included those associated with iron metabolism (TF, −0.39; hep, −0.21; Raida and Buchmann, 2009), immune regulation (IL11, −0.65; IL1R, −0.58; IL15, −0.53; Secombes *et al*., 2011) and inflammation (MMP13, −0.72; Krasnov *et al*., 2012).

**Figure 5:**
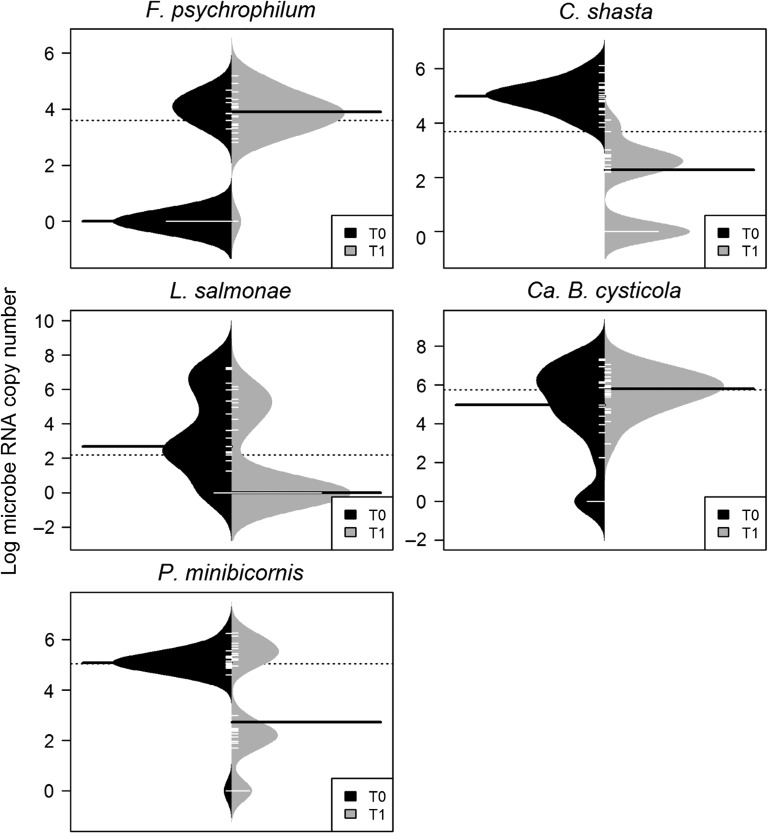
Beanplots of microbe productivity (log RNA copy number) at the time of gillnet capture (T0, *n* = 19; black) and 2 days following gillnet capture (T1, *n* = 22; grey) of Early Stuart sockeye in the Fraser River in Yale, BC. Polygons represent non-parametric density estimates, white bars represent total samples corresponding to RNA productivity, solid black bars represent the median productivity per time point (including negative detections) and dotted lines mark the overall median productivity. Significant differences in prevalence (*P* < 0.05) were identified for *Flavobacterium psychrophilum* and *Loma salmonae*, while *Ceratonova shasta* productivity differed between time points. Only microbes with sufficient total positive samples in one or both groups could be included in the analysis. Microbe productivities were measured from a small gill tissue biopsy (2–3 filament tips), normalized to 0.5 μg/μl of RNA per sample after RNA purification.

The prevalence of *F. psychrophilum* was lower at T0 than at T1 (*P* = 0.003), with no significant difference in productivity between time points (*P* = 0.515; Fig. 5). Conversely, prevalence of *L. salmonae* at T0 was higher than at T1 (*P* = 0.002), again with no significant difference in productivity (*P* = 0.093). The productivity of *C. shasta* was lower at T1 than at T0 (*P* < 0.001) with no significant difference in prevalence (*P* = 0.996). No significant differences in prevalence or productivity of *P. minibicornis* or *Ca.* B. cysticola were identified (*P*-values > 0.121), though the bimodal distribution of *P. minibicornis* at T1 suggests that a subset of individuals exhibited lower productivity.

### Factors influencing long-term survival to the spawning period

Microbe richness (total microbe species present) in gill tissue at capture was poor predictor of survival to the spawning period (GLM: *P* = 0.172). Co-infection was common both at capture and at death, with most fish carrying ≥3 microbe species. The multivariate classification tree model conducted on the gillnet-treated fish (20 s and 20 min) identified several variables reliably distinguishing ‘success’ and ‘failure’ to survive to the spawning period (Correct classification rate = 88%, Kappa = 75%, Monte–Carlo kernel-based *P* < 0.001). Plasma lactate was identified as the primary splitting criteria at 4.6 mmol/l, with low lactate individuals more likely to survive (85% correctly classified as success). Relative expression of Mx, an interferon-induced anti-viral protein, was identified as the secondary splitter among individuals with high lactate, with higher relative expression of Mx (≥−0.292) associated with failure (91% correct classification) and lower expression associated with success (83% correct classification). Eighty-nine percent of individuals with elevated plasma lactate (i.e. >4.6 mmol/l) and relatively high Mx expression (i.e. ≥−0.292) at the time of capture failed to survive beyond 20 dpt. Lactate and Mx were identified as the variables of greatest importance to decreasing node impurity (normalized quantiles: 100 and 88, respectively), as well as transferrin expression, plasma glucose, CD4 and interferon-α expression, and estimated percent lipid in muscle (normalized quantiles: >48).

### Factors associated with mortality

**Figure 6:**
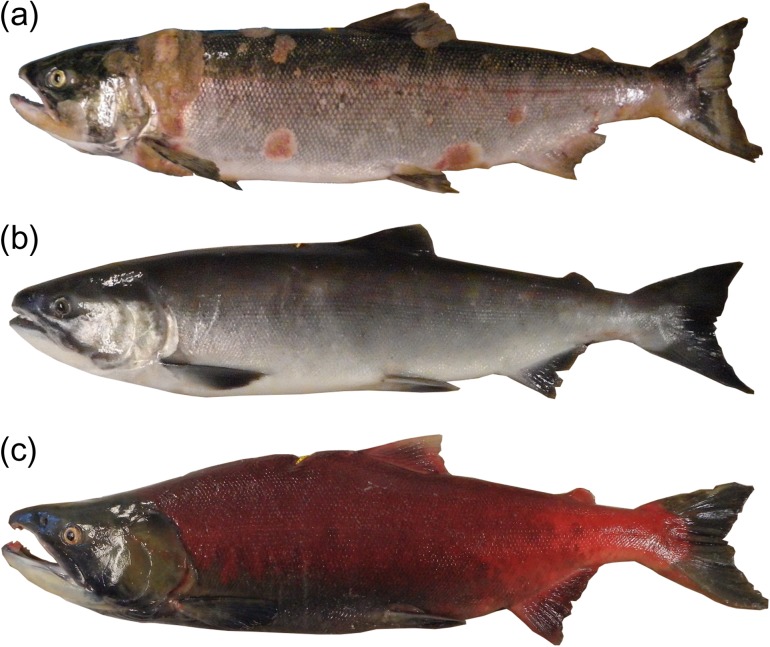
Three examples of Early Stuart sockeye exposed to experimental gillnet entanglement: (**a**) a prematurely moribund male showing severe necrosis and *Saprolegnia* spp. fungal infections, (**b**) a surviving male lacking secondary sexual characteristics and mild gillnet scarring posterior to the operculum and (**c**) a surviving male with well-developed secondary sexual characteristics and ventral gillnet scarring anterior to the dorsal fin.

**Figure 7:**
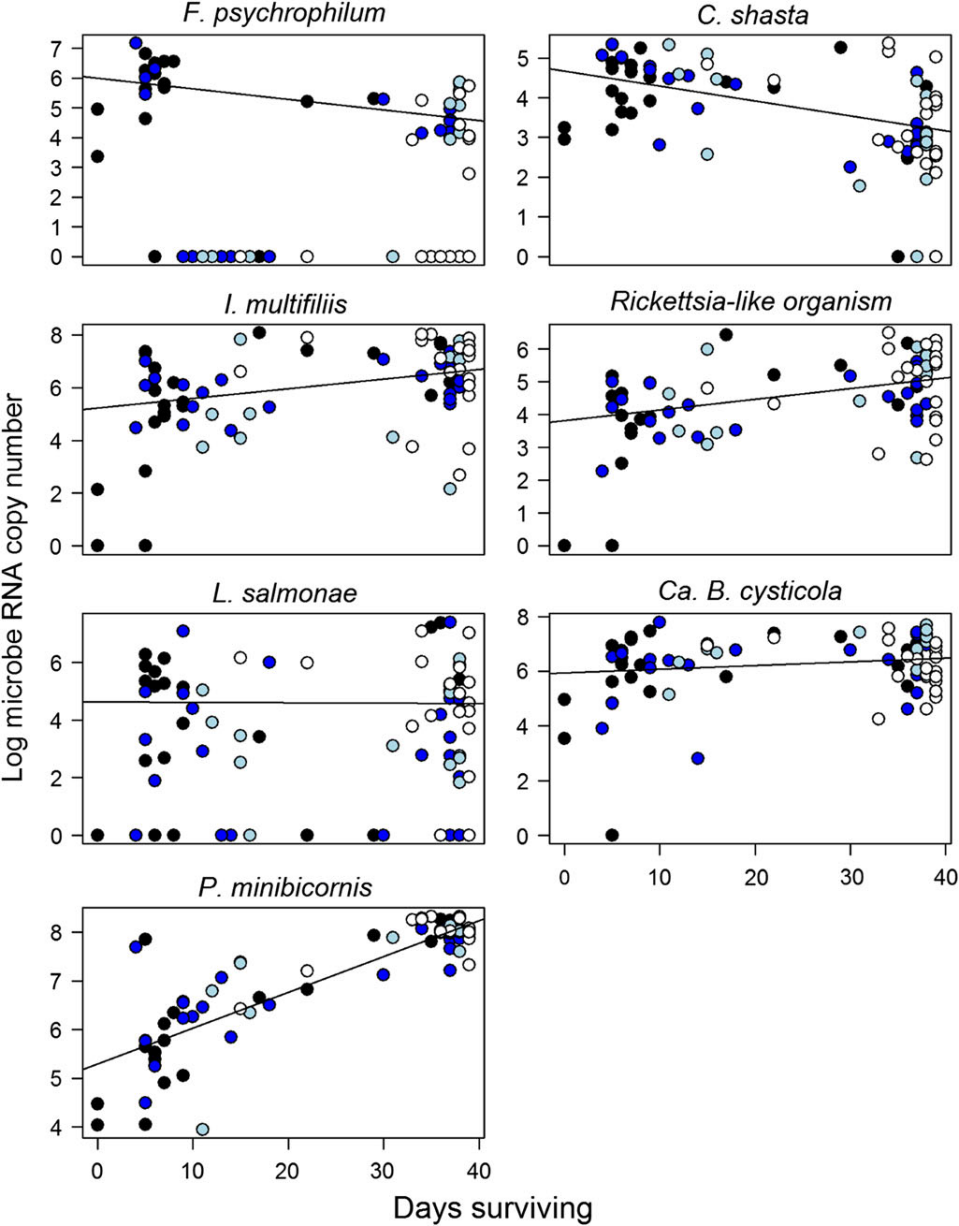
Relative productivity (log RNA copy number) of *Flavobacterium psychrophilum*, *Ceratonova shasta*, *Ichthyophthirius multifiliis*, Rickettsia-like organism, *Loma salmonae*, *Candidatus* Branchiomonas cysticola and *Parvicapsula minibicornis* as a function of days surviving for adult Early Stuart sockeye salmon. Each point represents the microbe burden of an individual at death; colour corresponds to treatment, with severe (20 min) entanglement in black, mild entanglement (20 s) in dark blue, biopsied controls in light blue and non-biopsied controls in white. Screening for microbes was conducted using a pool of aqueous phase from seven homogenized tissues including gill, liver, spleen, head kidney, heart, white muscle and brain (alternated every other individual). All relationships (linear models on positive detections) were significant (*P* < 0.05), except for *L. salmonae* (*P* = 0.97); model parameters can be found in the text.

Gross pathologies of many individuals at death included necrosis of skin, gill and muscle tissues and associated *Saprolegnia* spp. fungal infections in areas where the gillnet caused constriction or injury, such as behind the operculum (Fig. 6). However, not all individuals that died prematurely showed external signs of poor health. Many individuals failed to develop secondary sexual characteristics or ripen. Microbes measured in pooled tissues at death showed no significant change in prevalence with days surviving (logistic regression: all *P*-values > 0.05), likely due to the high prevalence of most microbes. Relative productivity of positively detected microbes, however, did show variation with time (Fig. 7, Table 5). *Flavobacterium psychrophilum* and *C. shasta* exhibited negative relationships with days surviving (*F. psychrophilum*: slope = −0.07, *r*^2^ = 0.250, *P* < 0.001; *C. shasta*: slope = −0.07, *r*^2^ = 0.195, *P* < 0.001). Positive relationships were identified for *I. multifiliis* (slope = 0.09, *r*^2^ = 0.141, *P* < 0.001), RLO (slope = 0.08, *r*^2^ = 0.168, *P* < 0.001) and *P. minibicornis* (slope = 0.16, *r*^2^ = 0.712, *P* < 0.001). The relationship of *Ca.* B. cysticola with days surviving was positive with a shallow slope (slope = 0.04, *r*^2^ = 0.074, *P* = 0.016), and *L. salmonae* showed no significant relationship (*P* = 0.967).

**Table 5:** Agreement between gill and pooled tissues in quantification of presence and relative productivities

Microbe	Abbreviation	Agreement between gill and pooled tissues (%)					Linear regression of positives			Breusch–Pagan test	
		Total agreement	Gill + Pool +	Gill −Pool −	Gill + Pool −	Gill −Pool +	*β*	*r*^2^	*P*	BP	*P*
*A. salmonicida*	ae_sal	99	1	98	0	1	NA	NA	NA	NA	NA
*Ca.*B. cysticola	c_b_cys	98	95	2	1	1	0.29	0.81	<0.01	28.5	<0.01
*C. shasta*	ce_sha	69	66	2	0	31	0.06	0.01	0.51	0.3	0.59
*C. salmositica*	cr_sal	98	0	98	0	2	NA	NA	NA	NA	NA
*D. salmonis*	de_sal	99	0	99	1	0	NA	NA	NA	NA	NA
*F. psychrophilum*	fl_psy	84	49	35	1	14	0.29	0.97	<0.01	3.2	0.08
*I. multifiliis*	ic_mul	94	75	19	1	5	0.45	0.74	<0.01	25.1	<0.01
*K. thyrsites*	ku_thy	98	11	87	0	2	0.22	0.29	0.13	0.6	0.43
*L. salmonae*	lo_sal	80	65	14	4	17	0.33	0.90	<0.01	25.7	<0.01
*P. minibicornis*	pa_min	99	99	0	0	1	0.23	0.55	<0.01	14.8	<0.01
*Rickettsia-like organism*	rlo	99	67	31	0	1	0.43	0.83	<0.01	16.9	<0.01
*S. destruens*	sp_des	95	0	95	2	2	NA	NA	NA	NA	NA
*T. bryosalmonae*	te_bry	94	8	86	0	6	0.43	0.44	0.11	0.5	0.46

Total agreement and sources of error in presence/absence of data are shown as percents; relationships between calculated productivities are shown as the slope (*β*), *r*^2^ and *P*-values from linear regression of gill and pooled values (predictor and response, respectively). Breusch–Pagan tests describe the heteroscedasticity of the linear relationships. Only positive values were included in the linear regression models.

The agreement between gill and pooled tissue positive microbe detections was relatively high overall, ranging from 69 to 99% total agreement (Table 5). *Ceratonova shasta* exhibited the lowest total agreement, primarily attributable to negative detection in gill and positive in pooled tissue, which was the most common source of disagreement (e.g. for *F. psychrophilum, L. salmonae, Tetracapsuloides bryosalmonae, I. multifiliis*), suggesting a potential for false negatives if only the gill tissue is used for screening. Coefficients of variation between estimated copy numbers of positively detected microbes were relatively consistent and all less than one. The lowest was found in *C. shasta* (slope = 0.06, *r*^2^ = 0.01, *P* = 0.51), primarily due to an outlier, though the relationship still showed high variability excluding the outlier (slope = 0.11, *r*^2^ = 0.22, *P* < 0.01). *Ichthyophthirius multifiliis* and RLO had the strongest and tightest relationships between methods (slopes > 0.43, *r*^2^ > 0.74, *P* < 0.01), likely due to their isolation within gill tissue and subsequently consistent dilution in the aqueous tissue pool with other tissue types following tissue homogenization. Increased variability in the relationship between the methods with increasing productivity was evident in five out of the nine microbes evaluated (*P*-values < 0.01); *C. shasta, K. thyrsites* and *T. bryosalmonae* exhibited too much variability overall or too few observations to ascertain heteroscedasticity and *F. psychrophilum* showed marginal non-significance (*P* = 0.08). Spearman's rank correlations revealed a strong correlation between the productivities of *I. multifiliis* and RLO (*r*_s_ = 0.92) and moderate correlation between the bacteria *F. psychrophilum* and *Ca.* B. cysticola (*r*_s_ = 0.41). All other correlations were low (*r*_s_ ≤ |0.35|).

Microbe prevalence in fish sacrificed at spawning grounds was similar to that of fish sacrificed riverside at Yale, BC and held in the laboratory (Table 2). Some microbes showed higher prevalence at spawning grounds, including *F. psychrophilum* (100% at spawning grounds, 55% among held fish), *T. bryosalmonae* (85% spawning grounds, 4% held) and *Sphaerothecum destruens* (23% spawning grounds, 1% held).

### Microbe productivity and host responses

**Figure 8:**
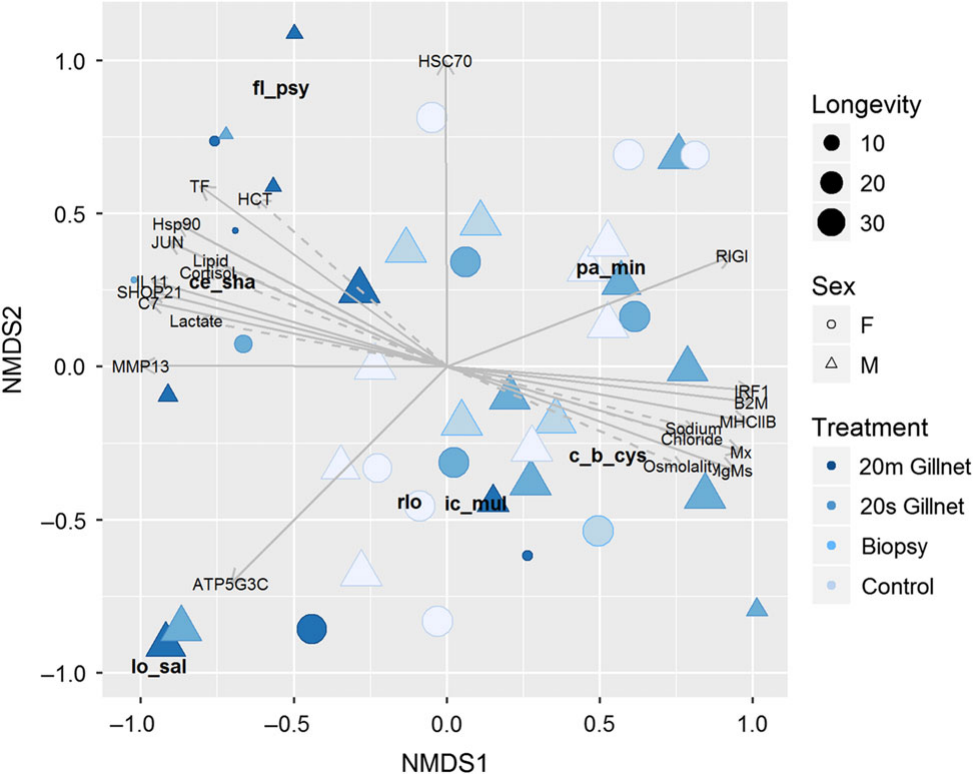
NNMDS plot of microbe productivities within the pooled tissues (gill, liver, spleen, heart, kidney, muscle, brain) of 42 Early Stuart sockeye. Vectors represent correlated (*P* < 0.10) host gene expression and plasma/muscle biomarkers of stress, condition and immunity. Shapes designate sex (filled circles = females, filled triangles = males) and colour represents the severity of handling and experimental gillnet treatment, with lightest to darkest as non-biopsied controls, biopsied controls, 20-s gillnet-treated, and 20-min gillnet-treated fish, respectively. The size of points represents longevity, with the largest points living the longest.

NMDS analysis of pooled tissue microbe data from individuals at death (including premature mortalities and survivors euthanized at the close of the spawning period) was successful in reducing the data into two-dimensional ordination (stress = 0.22, *P* = 0.001, Fig. 8). Fitted gill gene expression biomarkers significantly correlated with the ordination gradient at *P* < 0.01 for MHCIIB, JUN, IL11, B2M, TF, C7, IgMs and MMP13, at *P* < 0.05 for RIGI, Mx, SHOP21 and HSC70, and at *P* < 0.10 for HSP90, IRF1 and ATP5G3C. Clinical variables significant at *P* < 0.01 included plasma chloride and sodium, while cortisol, lactate, osmolality, haematocrit and muscle lipid were significant at *P* < 0.05. Longevity (days surviving) significantly correlated with the ordination gradient (*P* = 0.004), positively with NMDS1 and negatively with NMDS2, but treatment and sex did not (*P*-values = 0.13 and 0.82, respectively).

*Flavobacterium psychrophilum* and *C. shasta* were close in ordination space, negative on NMDS1 and positive on NMDS2. Expression of TF, C7, HSP90, JUN, SHOP21, IL11 and MMP13 as well as plasma cortisol, lactate, haematocrit and muscle lipid shared ordination space with *C. shasta* and *F. psychrophilum*. *Parvicapsula minibicornis* and *Ca.* B. cysticola were opposite in ordination space to *F. psychrophilum* and *C. shasta*, positive on NMDS1 and neutral on NMDS2, and associated with gene expression biomarkers RIGI (primarily *P. minibicornis*), IRF1, B2M, MHCIIB, Mx and IgMs, and the plasma variables sodium, chloride and osmolality, and host longevity. RLO and *I. multifiliis* were close in ordination space and to *Ca.* B. cysticola, falling neutral on NMDS1 and negative on NMDS2, directly opposite to HSC70 gene expression. *Loma salmonae* was isolated in ordination space from other microbes, loading negatively on both NMDS axes and positively associated with ATP5G3C expression.

## Discussion

Our results provide further evidence for delayed mortality following a capture stressor and illustrate influences of entanglement duration, sex and infectious disease processes on the survival of sockeye salmon in freshwater when exposed to a realistic temperature profile. Although severe entanglement (20 min) showed the greatest reduction in overall survival and days surviving, even a brief (20 s) encounter had a profound consequence, impacting both fish condition and survival. The 60% post-release mortality rate currently applied to Early Stuart sockeye released from Fraser River gillnet fisheries is similar to the raw mortality rates of 20 s and 20 min entangled fish (54 and 73% mortality, respectively, sexes combined), but is high after incorporating the effects of holding and biopsy (11–43% and 30–62% mortality, respectively). It should be noted, however, that entanglement treatments applied in the present study (20 s and 20 min) were on the low end of entanglement durations experienced by bycatch in actual gillnet fishery practices (e.g. set times may be >60 min; Farrell *et al*., 2001; DFO, 2002). Capture severity has previously been associated with enhanced mortality of released sockeye salmon in both field (Baker and Schindler, 2009; Donaldson *et al*., 2012; Baker *et al*., 2013) and laboratory settings (Gale *et al*., 2011), with more severe capture conditions and injuries correlating with greater physiological disturbances. Similar responses have also been identified in coho salmon (*Oncorhynchus kisutch*) caught in marine gillnet fisheries (Farrell *et al*., 2001; Buchanan *et al*., 2002). In the present study, the bulk of mortality following entanglement in freshwater did not occur until 5 days after the event, continuing at high rates for another 5–7 days, then subsiding in both treatment groups. The growing body of evidence for delayed mortality following capture in freshwater (Davis, 2002; Arlinghaus *et al*., 2007; Donaldson *et al*., 2010, 2012; Raby *et al*., 2012) and associated sex-specific effects (Martins *et al*., 2012b; Robinson *et al*., 2013; Donaldson *et al*., 2014; Gale *et al*., 2014) points to causal factors beyond short-term impacts, such as anaerobiosis or cardiovascular collapse, and instead towards interactions between the capture-related stress and infectious disease processes (Gilhousen, 1990; Raby *et al*., 2015) that can differ between sexes.

In the river, a 5–12 day delay would place mortality far upstream of the capture location, and certainly beyond the observation of the fishery, if released bycatch continued to swim upstream at estimated ground speeds (Rand and Hinch, 1998). For this reason and because this delay period is greater than the duration of most holding studies upon which management regulations are based (e.g. 24–48 h), current regulations may be underestimating mortality of released catch (Patterson *et al*., 2017a, 2017b). Additional factors including repeated capture in heavily fished regions, areas of challenging flows (e.g. Hells Gate) and rising river temperatures may exacerbate capture stress and mortality. Mortality in the present study spiked when temperatures were >15°C, which would correspond to when individuals would be moving through the Nechako and Stuart rivers. These high temperatures could affect the virulence of microbes carried and encountered by the host as well as host resistance (Altizer *et al*., 2013), contributing to pre-spawn mortality at the spawning grounds (Macdonald *et al*., 2012). Although temperature is generally inversely correlated with discharge in the Fraser River (Patterson and Hague, 2007; Macdonald *et al*., 2010), inclusion of multiple temperature or flow treatments would help to elucidate the roles of temperature and flow as compounding factors of fisheries capture stress.

Two control groups were included in the present study to assess the effects of holding and additional handling on survival. Mortality was high among biopsied controls, with females experiencing almost twice the mortality of males, whereas non-biopsy controls survived extremely well. This same biopsy technique has been applied in field telemetry studies on sockeye in the Fraser River and has been associated with similar or lower survival to spawning grounds (Donaldson *et al*., 2010, 2011, 2012), but generally higher survival in marine or coastal waters (Cooke *et al*., 2005; Martins *et al*., 2012b). Consistently lower survival and fewer days surviving (6–7 days) for females in the present study could be attributed to the biopsy-sampling procedure or entanglement. All gillnet-treated fish were biopsied, and biopsied controls as well as gillnet-treated fish exhibited sex-specific mortality. Controlling for the effects of handling in experiments is difficult as all subjects must be tagged or handled for study. Because the sample size for our biopsied controls was low relative to other treatment groups, further investigation of the biopsy effect is needed before firm conclusions can be drawn as to its independent impact on adult salmon survival and physiology in freshwater. Collectively, these findings suggest high sensitivity to any level of handling during river migration, especially for females.

Physiological and infectious disease-associated effects of capture and handling were apparent immediately and in the days and weeks following entanglement. Enhanced anaerobic activity following entanglement, especially when compounded by an intracellular immune response in the gill, was predictive of mortality. Because multiple infections were common at entanglement and at death, microbe species richness alone was a poor predictor of host survival. Fish that died prior to the spawning period showed elevated productivity of *F. psychrophilum* and *C. shasta* at death relative to survivors. These microbes were associated with enhanced indicators of stress and injury and diminished immune responses in dying fish. Conversely, productivity of the myxozoan parasite *P. minibicornis* and several other microbes was positively associated with longevity and a strong immune response. Regulation of stress and immune biomarkers and blood properties at death therefore differed depending on microbe community composition, which was strongly associated with host longevity. This pattern suggests alternate responses to infections or opportunistic strategies of certain microbes that may influence post-release survival. The mechanisms of post-release mortality likely depend not only on the severity of the capture event (and likely sex), but on the condition and response of individuals prior to, during, and following capture, which are influenced by or associated with infectious disease processes.

### Short-term responses to capture and predictive factors of mortality

Immediate and short-term responses to capture involved aspects of primary, secondary and tertiary features of the stress response (Barton and Iwama, 1991). High levels of plasma chloride following capture indicated osmoregulatory imbalance associated with a stress response, and has been previously documented in adult sockeye in freshwater stressed by both warm water (Jeffries *et al*., 2012a) and a simulated gillnet entanglement (Donaldson *et al*., 2012). Elevated plasma cortisol was associated with shifts in microbe community structure following capture and altered gene regulation in the gill suggestive of immunosuppressive effects. Reproductive hormones were also suppressed in the days following capture and many fish failed to develop secondary sexual characteristics, a phenomenon also observed in Alaskan sockeye salmon with injuries indicating escape from gillnet fisheries (Baker *et al*., 2013). Although delayed maturation observed in the present study could be due to either capture or holding (Patterson *et al*., 2004), this finding warrants further investigation into how salmon recover from an acute stressor under chronically stressful conditions. Chronic stressors such as high river temperature or discharge have been shown to reduce the reproductive output of Early Stuart sockeye salmon (Braun *et al*., 2013). Our results together with previous findings suggest that these animals are poorly equipped to manage the metabolic demands of long (>20 min) and sometimes even brief (20 s–3 min) capture durations, with potential carryover effects on reproduction (O'Connor *et al*., 2014).

Both immediate responses to and condition during capture—specifically elevated plasma lactate levels (i.e. anaerobic exercise) and Mx gene expression in gill—were predictive of survival to the spawning period of Early Stuart sockeye salmon. The importance of maintaining aerobic metabolism in bycatch is evident from the high plasma lactate levels of fish destined to die prior to the spawning period (even after mild entanglement), utilizing anaerobic respiration during entanglement and unable to clear metabolites (Jain and Farrell, 2003). Strenuous exercise and air exposure are common occurrences in bycatch scenarios, and both contribute to elevated lactate levels measured in blood plasma (Gale *et al*., 2011; Cooke *et al*., 2013). Although microbe productivity did not emerge as an important predictor of survival, expression of Mx—an interferon-induced antiviral protein—in gill was included in the predictive model. Elevated Mx expression has been associated with mortality of juvenile (Jeffries *et al*., 2014a) and adult sockeye (Miller *et al*., 2011), though the specific mechanisms driving this correlation are unknown. Viral infection was not identified as prevalent or correlated with Mx expression in this experiment, and *F. psychrophilum* and *C. shasta*, though identified as potential pathogens in this study, were not correlated with Mx expression. Despite the comprehensiveness of our screening approach, an unknown viral agent may have been present within these fish. Alternatively, expression patterns may be a relic of cleared, latent, or anticipated viral infection: freshwater-resident *Oncorhychus mykiss* have shown overexpression of antiviral transcripts relative to out-migrating smolts, suggesting a modulation of immune defences towards viral pathogens in freshwater (Altizer *et al*., 2011; Sutherland *et al*., 2014). Enhanced expression of immune transcripts, including both cellular and humoral responses, have been documented in sockeye salmon during the spawning migration, corresponding to shifts in environmental and biotic factors (Evans *et al*., 2011). In the present study, relative expression of Mx at death was positively associated with days surviving, which may indicate modulation of this gene's expression as part of the senescence trajectory of Pacific salmon. This temporal confounding would explain the lower Mx expression levels found in premature mortalities at death relative to survivors, with values more similar to those measured at entanglement. Regardless of its mechanism, elevated Mx expression in the presence of high plasma lactate levels was associated with an 85% chance of premature mortality, demonstrating an association between these factors that may suggest synergistic or additive effects on the likelihood of host survival.

### The role of microbes in post-release mortality

Large-scale animal migrations have been identified as potential mitigation measures for disease epidemics that may otherwise greatly impact population dynamics (Altizer *et al*., 2011). Infectious agents therefore likely play a measurable role in determining survival of salmon during spawning migrations. This would especially be true for semelparous Pacific salmon with a fixed energy budget to fuel their migration to natal waters where they will die soon after spawning (Rand *et al*., 2006). Alterations to historic migratory conditions, such as increased anthropogenic activities like fishing or hydrologic changes, may offset the balance of established host–parasite relationships by lessening the effectiveness of current life history strategies in attenuating disease proliferation within and among individuals. Premature mortality of adult spawners could therefore result from a number of related causes including an upset to the community dynamics of microbes carried by the host, diminished host resilience, enhanced susceptibility to infection, or a combination of these phenomena. Distinct host responses to microbe infections may influence survival following capture, as microbe community structure at death and host gene regulation were strongly associated with longevity in the present study. Two microbes in particular, *F. psychrophilum* and *C. shasta*, exhibited pathogenic characteristics, with short- and long-term changes in their prevalence or productivity and concurrent shifts in host physiology.

Short-term increases in *F. psychrophilum* prevalence following gillnet capture may signify a means of enhanced transmission among sockeye salmon *en route* to spawning grounds, potentially increasing the influence of this bacteria in intensely fished regions. *Flavobacterium psychrophilum* was present in all gillnet-treated fish at entanglement. Because the collection and treatment nets were not disinfected between fish, *F. psychrophilum* could have been transferred *via* the net—a possibility that certainly exists for a real fishery. Captivity may have artificially increased the prevalence of *F. psychrophilum* following capture, but may also reflect transmission dynamics that would occur naturally among migrating individuals packed in resting pools or in pre-spawning aggregations in the river (Gilhousen, 1990). This bacteria, which has been shown to increase in prevalence towards spawning grounds (Miller *et al*., 2014), excretes a psychrophilic protease causing lesions and necrosis in affected tissues and has been found to suppress humoral immune defences (Barnes and Brown, 2011). Indeed, we noted lesions on many moribund fish and aspects of the complement system's membrane attack complex (C3, C4B) showed lower expression following capture. Prevalence in this study could also be considered a proxy for productivity as low RNA copy numbers, either below qPCR detection limits or in isolated regions of the gills not sampled, would produce negative detections; the likelihood of false negatives would be expected to decrease as infections intensify. Productivity of *F. psychrophilum* in the present study was also associated with decreased plasma ion levels (sodium, chloride) in both moribund and surviving fish. Osmoregulatory failure is predictive of mortality in Pacific salmon, with plasma ion and osmolality levels dropping by 20–40% prior to death (Jeffries *et al*., 2011). Our results demonstrate a link between the productivity of *F. psychrophilum* and osmoregulatory failure, which may result from opportunistic enhancement of *F. psychrophilum* in a compromised host or loss of ion homeostasis due to tissue damage caused by *F. psychrophilum* (Barnes and Brown, 2011).

Lower productivity of *C. shasta* in the days following capture could be attributed to the life cycle of this myxozoan parasite, as spores enter the host through the gills but migrate to the gut to mature (Bartholomew, 1998). Whether such pathogenesis could be initiated by acute stress is unknown; chronic thermal stress has been positively associated with ceratomyxosis and mortality of Klamath River salmon, but likely *via* enhanced spore densities in the river (Ray *et al*., 2012). An intermediate polychaete host releases infectious *C. shasta* spores into the river as adult salmon begin their upstream migration (Bartholomew *et al*., 1997); peaks or lulls in spore densities and the rate at which salmon pass through the lower river influence ‘dosage’ and subsequent infection intensities (Stocking *et al*., 2006). Throughout the period of collection (2 days), fish were randomized among treatment groups to reduce any time-associated bias in microbe productivities. As we did not include intestine in our tissue screen, *C. shasta* productivity measured here does not account for changes in the gut, which may have provided further information relative to its pathogenicity in Fraser River sockeye salmon. Furthermore, disagreement between detections in gill and pooled tissues suggests that pathogen screening *via* gill biopsy may underestimate the infection intensity of *C. shasta*. However, because *C. shasta* infects the host *via* the gill, this tissue provides some insight into infectious load, a key factor for host survival (Stocking *et al*., 2006). The presence of *C. shasta* within the pooled tissues (including gill) of all prematurely morbid gillnet-treated fish points to reduced survival of infected hosts.

*Flavobacterium psychrophilum* and *C. shasta* were most productive in the earliest mortalities at death and were associated with similar responses in both gill gene expression and plasma indices of host stress and immunity. This finding suggests either a mutualistic relationship between these microbes, similarly opportunistic productivity in dying hosts, or equally pathogenic effects. Although our terminal analysis did not identify a significant relationship of gillnet treatment with microbe community dynamics, most premature mortalities were experimentally entangled. Among dying fish, productivities of *F. psychrophilum* and *C. shasta* (to a lesser extent) negatively or neutrally correlated with relative expression of most intra- and extracellular immune genes (e.g. IgMs, Mx, MHCIIB, B2M and IRF1), which may illustrate a relationship between immunosuppression and microbe productivity. This response may indicate an inability of the host to maintain an adequate defence against these pathogens in the days or weeks following capture. Downregulation of several immune response factors in the gill transcriptome of moribund fish has previously been described in adult sockeye salmon exposed to thermal stress, suggesting potential immunosuppression-linked mortality in stressed fish (Jeffries *et al*., 2012b). The few immune genes that were positively correlated with *F. psychrophilum and C. shasta* indicated an innate pathogen defence, including compromised epithelial integrity and inflammation (MMP13, IL11;Trepicchio *et al*., 1996; Wang *et al*., 2005), pathogen lysis (C7;Gonzalez *et al*., 2007) and iron metabolism (TF; Raida and Buchmann, 2009). Enhanced expression of MMP13 and IL11 have also been associated with imminent mortality of Chilko sockeye smolts at the start of seaward migration (Jeffries *et al*., 2014a); because *F. psychrophilum* was not prevalent and *C. shasta* (as well as other agents included here) not measured in Chilko smolts, such gene regulation may be part of a more universal response to handling stress.

Other microbes such as *I. multifiliis*, RLO, *Ca.* B. cysticola and *P. minibicornis* showed increasing productivity with days surviving and more positive associations with biomarkers of immunity at death. This association indicates an immune response from the host late in the migration rather than sooner (i.e. stronger immune responses among surviving fish during the spawning period). Mortality associated with these microbes would therefore be more likely to occur at spawning grounds instead of *en route* and thus not necessarily be attributable to capture but rather senescence. However, it is possible that some individuals that died prematurely had a decreased threshold for productivity of these microbes (Bradford *et al*., 2010). Their negative association with stress and wound healing biomarkers does not support an opportunistic pathogenicity in immune-compromised fish, but rather an elicited immune response by apparently non-stressed individuals in association with high microbe productivities. *Ichthyophthirius multifiliis* and RLO were clustered closely on the NMDS and their productivities were highly correlated, which supports an endosymbiosis previously identified between this ciliate and *Rickettsia* bacteria (Sun *et al*., 2009). Expression of the heat shock cognate 70 (HSC70), a molecular chaperone that has shown variable responses to stressors (Boone and Vijayan, 2002), was inversely correlated with *I. multifiliis* and RLO on the NMDS gradient. Although downregulation of this gene has been noted in salmon at spawning grounds (Miller *et al*., 2009), the relationship of HSC70 with longevity was weak relative to its correlation with *I. multifiliis* and RLO productivities. The mechanism for this inverse relationship warrants further investigation. Productivity of *I. multifiliis*, RLO, *Ca.* B. cysticola and *P. minibicornis*, as well as the expression of antibody (IgMs), intracellular (Mx, B2M), extracellular (MHCIIB) and antiviral (IRF1, RIG1) host genes, plasma osmolality and ions were all positively associated with host longevity. Because longevity was impacted by gillnet entanglement and handing, capture stress cannot be excluded as a potential modifier of infectious agent communities and host responses.

*Loma salmonae*, a microsporidian parasite known to infect sockeye salmon in BC (Shaw *et al*., 2000), showed lower prevalence in the days following capture. A bimodal distribution of productivities was present at collection but absent in the days following with more negative detections; low productivities could therefore have been reduced below detection limits while higher productivities remained detectable. The rupture of xenomas (spore aggregations in host cells) in gill lamellae and subsequent spore release and dispersal (Rodriguez-Tovar *et al*., 2011) could reduce prevalence by decreasing the probability of detection in gill. Elevated expression of the inflammation-associated collagenase MMP13 gene in gill could be attributed to xenoma rupture or, alternatively, *F. psychrophilum* infection (Kent and Speare, 2005; Langevin *et al*., 2012) or gillnet injury. *Loma salmonae* displayed unique characteristics at death among the microbes evaluated, showing no significant changes in productivity with time in morbid fish. Expression of ATP5G3C was associated with *L. salmonae* productivity (and *I. multifiliis* and RLO to a lesser degree), suggesting enhanced cellular energy needs in the gills of infected fish, potentially due to ruptured xenomas and damage to gill lamellae (Kent and Speare, 2005). This parasite was not strongly associated with survival of Early Stuart sockeye in this study, though productivity measured in severely gillnetted fish at death was generally higher than in other groups (data not shown). A negative impact of *L. salmonae* infection on the survival of adult Chilko sockeye tagged in the marine environment (Miller *et al*., 2014) may suggest that associated losses are stock-specific or occur closer to the river mouth than where our fish were collected.

Collectively, these changes in microbe productivity and prevalence suggest a dynamic community and warrant further investigation into their natural trajectories throughout the entire migration period. Microbe prevalence in the laboratory was similar to that measured at spawning grounds, though sample sizes were limited. However, some pathogenic microbes including *F. psychrophilum* and *T. bryosalmonae* (etiological agent of proliferative kidney disease; Longshaw *et al*., 2002) were more prevalent at spawning grounds; fish held in the relatively sterile laboratory environment may have therefore been protected from additional infectious agents affecting fish in the river, which could have enhanced their survival (Benda *et al*., 2015). Causal relationships between microbe infections and changes in host responses have yet to be established and will require an improved understanding of the baseline trajectories of microbe productivities and host responses over time in wild fish.

### Sex-specific differences

Females showed more susceptibility to impairment imposed by handling stress, with unique physiological changes relative to males and consistently greater (and earlier) mortality among biopsied controls and gillnet-treated fish. Given that biopsied controls experienced high sex-specific mortality, extrapolating this sex effect beyond experimental handling is speculative. However, sex-specific effects have been repeatedly documented in response to capture stress with respect to survival and physiological effects (Martins *et al*., 2012b; Robinson *et al*., 2013; Donaldson *et al*., 2014; Gale *et al*., 2014). Martins *et al*. (2012a) noted, for example, that females exhibited lower survival when compounding stressors (e.g. temperature and hydraulic challenges) were acting simultaneously. Individuals in the present study were exposed to a similar thermal experience to those travelling in the river, which corresponds to an increase in temperature with time in the first 1–2 weeks of the migration until thermal refugia can be accessed (Rand and Hinch, 1998). Female sockeye salmon have been shown to perform poorly when exposed to chronic high temperatures, with lower survival among females held at 19°C relative to those held at 13°C and overall lower survival relative to males (Jeffries *et al*., 2014b), but few transcriptomic differences between sexes. Sex-specific differences in gene expression were identified at the time of collection in the present study, with females exhibiting more consistently elevated expression of immune-related gene transcripts in gill than males, suggesting that females may differ in their response to migratory stress in the river. As a secondary stress response, short-term differences in glucose levels following capture were also sex specific, pointing to a stress-related release of glucose needed to support aerobic respiration in females that is absent in males (Barton, 2002), though possibly due to holding rather than capture (Donaldson *et al*., 2011). Causal factors for higher female mortality during river migration have yet to be fully characterized.

## Conclusions

Post-release mortality of adult salmon has repeatedly been shown to be context specific and dependent on numerous factors (Raby *et al*., 2015). The mechanisms of mortality following capture are therefore complex and include internal and external influences (Fenkes *et al*., 2016). In addition to previously identified factors, such as temperature and sex, our results support a role for capture severity and infectious disease processes in impacting the survival of released bycatch in freshwater. Anaerobic activity in concert with an intracellular immune response was associated with decreased long-term survival to the spawning period. The benefits of removing bycatch from the net quickly and under-water, though already embraced by some fishers as best practices, should be widely disseminated among user groups. The degree to which temperature impacts post-release mortality rates deserves further investigation, as the majority of premature mortality occurred 5–12 days after entanglement, when temperatures were at their highest. Early Stuart sockeye navigate a balance of challenging migratory conditions, when the timing of river entry coincides with high flows often accompanied by lower temperatures or high temperatures with generally more manageable flows (Morrison *et al*., 2002). Projected climate-associated changes to Fraser River hydrology include a shift in the timing of the spring freshet to earlier in the year, which would result in elevated river temperatures during the historical migration period of Early Stuart sockeye (Morrison *et al*., 2002; Patterson *et al*., 2007). These changes have been associated with elevated levels of both *en route* and pre-spawn mortality, likely due to stress and disease processes as well as enhanced energy consumption by migrating sockeye salmon utilizing a fixed energy budget (Gilhousen, 1990; Macdonald, 2000; Crossin *et al*., 2008; Martins *et al*., 2012b; Fenkes *et al*., 2016).

The observed delay in post-release mortality not only has implications for how managers assign mortality rates to release fisheries, but alludes to its causes. Interactions between anaerobic activity, changes in microbe community dynamics, and shifts in local (gill gene expression) and systemic (plasma hormones and metabolites) stress and immune responses affect the chances of survival following capture. Although our experimental design prohibited direct comparison of treatment groups at death, most fish exposed to severe gillnet entanglement died prematurely, showing higher productivities of *F. psychrophilum* and *C. shasta* and demonstrating response profiles distinct from survivors. A more complete understanding of the natural trajectory of infectious agent productivities and host responses will improve our understanding of how stressors modulate these factors throughout migration and should be a top priority for future research. Our findings offer insight into the linkages between physiology, infectious agents and post-release survival following gillnet capture, thereby improving our understanding of the mechanisms contributing to mortality of released catch.
